# Developing a framework to inform scale-up success for population health interventions: a critical interpretive synthesis of the literature

**DOI:** 10.1186/s41256-020-00141-8

**Published:** 2020-04-29

**Authors:** Duyen Thi Kim Nguyen, Lindsay McLaren, Nelly D. Oelke, Lynn McIntyre

**Affiliations:** 1 Department of Economics, Faculty of Business, University of New Brunswick, 100 Tucker Park Road, P.O. Box 5050, Saint John, New Brunswick E2L 4L5 Canada; 2 School of Epidemiology and Public Health, Faculty of Medicine, University of Ottawa, 600 Peter Morand Crescent, Ottawa, Ontario K1G 5Z3 Canada; 3 New Brunswick Health Research Foundation, 10 Knowledge Park Drive, Fredericton, New Brunswick E3C 2M7 Canada; 4 Department of Community Health Sciences, Cumming School of Medicine, University of Calgary, TRW3, 3280 Hospital Dr. NW, Calgary, Alberta T2N 4Z6 Canada; 5 O’Brien Institute for Public Health, University of Calgary, TRW3, 3280 Hospital Dr. NW, Calgary, Alberta T2N 4Z6 Canada; 6 School of Nursing, Faculty of Health and Social Development, University of British Columbia, 3333 University Way, Kelowna, British Columbia V1V 1V7 Canada

**Keywords:** Population health intervention, Scale-up, Framework, Critical interpretive synthesis, Readiness

## Abstract

**Background:**

Population health interventions (PHIs) have the potential to improve the health of large populations by systematically addressing underlying conditions of poor health outcomes (i.e., social determinants of health) and reducing health inequities. Scaling-up may be one means of enhancing the impact of effective PHIs. However, not all scale-up attempts have been successful. In an attempt to help guide the process of successful scale-up of a PHI, we look to the organizational readiness for change theory for a new perspective on how we may better understand the scale-up pathway. Using the change theory, our goal was to develop the foundations of an evidence-based, theory-informed framework for a PHI, through a critical examination of various PHI scale-up experiences documented in the literature.

**Methods:**

We conducted a multi-step, critical interpretive synthesis (CIS) to gather and examine insights from scale-up experiences detailed in peer-reviewed and grey literatures, with a focus on PHIs from a variety of global settings. The CIS included iterative cycles of systematic searching, sampling, data extraction, critiquing, interpreting, coding, reflecting, and synthesizing. Theories relevant to innovations, complexity, and organizational readiness guided our analysis and synthesis.

**Results:**

We retained and examined twenty different PHI scale-up experiences, which were extracted from 77 documents (47 peer-reviewed, 30 grey literature) published between 1995 and 2013. Overall, we identified three phases (i.e., Groundwork, Implementing Scale-up, and Sustaining Scale-up), 11 actions, and four key components (i.e., PHI, context, capacity, stakeholders) pertinent to the scale-up process. Our guiding theories provided explanatory power to various aspects of the scale-up process and to scale-up success, and an alternative perspective to the assessment of scale-up readiness for a PHI.

**Conclusion:**

Our synthesis provided the foundations of the Scale-up Readiness Assessment Framework. Our theoretically-informed and rigorous synthesis methodology permitted identification of disparate processes involved in the successful scale-up of a PHI. Our findings complement the guidance and resources currently available, and offer an added perspective to assessing scale-up readiness for a PHI.

## Background

To date, efforts to scale-up population health interventions (PHI) have been met with variable success [1–4]. To help guide successful scale-up of a PHI, practitioners may draw upon evidence-based and theory-informed frameworks. In this paper, scale-up refers to the deliberate process of enhancing the impact of an effective intervention [5]. A PHI is defined as a discrete set of actions (e.g., policy, program) that impact a number of people by attending to underlying conditions (i.e., social determinants) of health risk, thereby improving population health and reducing health inequities [6].

Within the past twenty years, the number of resources to support the practice of scale-up has grown. ExpandNet, a network of public health professionals that was developed in collaboration with the World Health Organization (WHO), has published several important resources, including the ExpandNet conceptual framework [5], which helps facilitate the strategic planning and management of the scale-up process. Subsequent ExpandNet resources include practical guidance for scaling up health innovations [7], steps for developing a scale-up strategy [8], and scale-up planning [9]. Other notable frameworks include Cooley and colleagues’ Scaling Up Management framework [10–12], Yamey’s framework regarding scale-up success factors [13], and Greenhalgh et al.’s framework about predicting and evaluating scale-up [14], to name a few.

Scale-up is an emerging field. To advance the science and practice of scale-up, there are several knowledge gaps that warrant further investigation. For example, the scale-up literature has been criticized for simplistic conceptualizations of the process [3], scarcity of theory [15, 16], and lack of focus on sustainability [17]. In addition, while some studies have adopted a theoretical lens to examine scale-up, such as diffusion of innovation [5, 18] and complex adaptive systems [3, 19], one theory that has received little attention, but that would complement and add value to our understanding of the scale-up process, is the organizational readiness for change theory. Readiness has been recognised as integral to successful change (e.g., scale-up), and insufficient readiness may be responsible for as many as half of all unsuccessful change attempts made by large-scale organizations [20]. According to this change theory, readiness requires the organization’s *willingness* to change and *capability* for change implementation; the latter depends on knowing the necessary actions for change, sequence of these actions, and required resources and capacities [20]. Thus, to assess readiness, the scale-up team (e.g., scale-up practitioners, decision makers) must have prior knowledge of the entire process that is required for change (i.e., scale-up success). To date, studies that have examined scale-up readiness have examined readiness against a set of benchmarks indicators specific to newborn survival interventions [21] or by conducting interviews and consensus building workshops regarding nutrition interventions [22, 23]. Because PHIs are considered complex and may address a variety of different target population and health issues, an alternative approach to assessing readiness for a PHI may include examining one’s own collective capacity in relation to what is needed for a successful scale-up. Drawing on the organizational readiness for change theory, the scale-up team may assess readiness for scale-up success by understanding the necessary actions for scale-up success, including the general sequence of these actions and the capacities required, and then comparing it to one’s own unique circumstance.

We sought to build upon existing scale-up frameworks, and address several literature gaps, by developing a practical, validated, evidence-based and theory-informed framework that may aid in successfully scaling-up a PHI. To assist in scale-up readiness assessment, our intent is to provide a realistic depiction of the pathway of successfully scaling-up a PHI, by examining a diverse collection of contemporary, real-life, scale-up experiences and shedding light on the complexities and nuances of each key action identified in the scale-up process. Framework development was guided by theories relevant to scale-up (i.e., organizational readiness for change, complex adaptive systems, and diffusion of innovation), to help identify and understand the key actions necessary for scaling-up a PHI.

The development of this framework was conducted in two stages. In this paper, Stage 1, we aimed to identify the foundational content of our framework by critically examining and drawing new insights from various scale-up experiences across the globe. To advance our understanding of the entire scale-up process (as recommended by the organizational readiness for change theory), we sought to answer the following research question: *How is scale-up of a PHI successfully implemented and sustained?* Stage 2 of our overall study includes the validation and practical assessments of our working framework, which is reported elsewhere [24].

## Methods

We employed the critical interpretive synthesis (CIS) method to examine the implementation and sustainability of scale-up, across a wide range of PHIs globally. The CIS is well-suited for our goal because it permits a critical analysis of the literature [25] for an exploratory research topic that is broad, not precisely defined, and spans a diverse and complex body of work [26]. The purpose of the CIS is to maximize contributions towards conceptual and theoretical development, not an exhaustive summary of all available data [25], and the resulting critical synthesis is ideal for developing the foundational content for a framework.

Rather than a focus on comprehensive identification and inclusion of all relevant literature, as would be the case in a conventional systematic review, the focus in the CIS is to gather a rich sample of the diverse literature until saturation has been reached (i.e., until new data was repetitive and failed to add significant insight or interpretive value) [25]. Many scholars caution against the use of a rigid systematic literature review process when exploring complex, broad topics, as these topics typically do not have universal definitions used across the literature, and thus lack specific search terms and inclusion/exclusion criteria [27–29]. Consequently, the CIS approach is ideal for our paper because it promotes flexibility and the use of an iterative, interactive, and recursive approach; allowing the researcher to refine and adapt their protocol as they gain a deeper understanding of the literature [25, 30, 31]. A full description of the CIS key processes and characteristics is provided in Table 1.

**Table 1:** Key processes and characteristics of a critical interpretive synthesis

Key Process or Characteristic	Description
Purpose	To conduct a critical analysis and generate new insights of a topic by examining a broad base of relevant literature.
Process	CIS rejects the “staged” approach to the literature review process and instead supports an iterative, interactive, and recursive approach which recognizes the need for flexibility and reflexivity. Searching, sampling, critiquing, reflecting, and analysis may occur in tandem and/or iteratively. Due to the interpretive process, it is acknowledged that some aspects may not be auditable or reproducible. A precise protocol for CIS is not offered due to the acknowledgement of the “authorial voice”.
Synthesis question	This approach is ideal for synthesis topics that may not be precisely bounded or clearly defined; as the synthesis progresses a more precise definition may develop. Similarly, the review question may evolve and become refined as the synthesis progresses.
Search strategy	Various literature sources may be utilized, including literature databases (peer-reviewed, grey), reference citation, snowballing/review of bibliography, hand searching, expert consultation, and author contacts. Beginning with a broad strategy, the strategy may evolve organically.
Sampling	Eligible studies may include empirical/theoretical literature, editorials, commentaries, and reviews. Inclusion criteria can be flexible and to some extent emergent. The purpose of sampling is to be extensive, but not comprehensive, therefore study selection may include purposive and theoretical sampling. Ongoing selection of potentially relevant literature is informed by reflection and emerging concepts. The intent is to sample literature that will maximize contributions towards conceptual and theoretical development.
Critical appraisal	Depending on the purpose of the review, methodological quality assessment is optional. If the review includes various types of data it may not be feasible to assess methodological rigour consistently as there is no single tool that may be used across all types of studies. CIS suggests greater emphasis should be placed on critiquing throughout the CIS approach rather than just critical appraisal during the sampling phase.
Data extraction	Use of a formal/standard data extraction form is optional.
Coding	Codes for the data are derived from the literature (i.e., inductive).
Analysis	Data analysis includes components of critique, reflection, interpretation, development of new concepts, and integration. Synthesis goes beyond summarization and includes the critical examination, interpretation, and generation of new insights.
Results	CIS leads to the generation of a synthesizing argument (e.g., theory, framework), a critically informed analysis that provides new insights by identifying relationships within and/or between existing constructs in the literature and ‘synthetic constructs’ (new constructs generated through synthesis). The synthesizing argument is grounded in the literature and formed by the process of integrating evidence from across the studies.

Note: Adapted from Dixon-Woods et al. (2006) & Entwistle et al. (2011)

### Search and selection strategy

Between November 2013 and September 2014, we searched for English-language literature regarding PHI scale-up. We sought a diverse, rich sample of highly relevant studies. Accordingly, we searched specific sources that were relevant to scaling-up. First, we searched specialized databases including ExpandNet, Health Systems Evidence, World Health Organization Library (WHOLIS), and Campbell Collaboration Library. Keyword and phrase searches within titles and abstracts were conducted for electronic databases (see Additional file 1 for search terms used). Second, we hand-searched select journals (i.e., Implementation Science; Health Policy and Planning, Bulletin of the World Health Organization). Third, we consulted scale-up and PHI experts for additional literature. Fourth, we screened reference lists of key articles (i.e., [13, 16]), as well as other articles that have cited these key articles (according to Google Scholar). Fifth, we searched websites that potentially contained literature regarding the scale-up of a PHI (see Additional file 2).

For some PHIs, the descriptions of the PHI, context, and scale-up attempt were documented across several articles; thus, the PHI was the unit of analysis. A scale-up attempt of a PHI was considered eligible for CIS inclusion if:

i. There was compelling evidence the PHI impacted a number of people and had the potential to improve health and reduce health inequities by addressing one or more social determinants of health [32];
ii. An explicit attempt was made to implement and/or sustain scale-up of a PHI; and
iii. The study provided substantive experiential evidence regarding the scale-up process.

Consistent with the CIS approach, purposive sampling from eligible studies was driven by conceptual relevance and no studies were excluded on methodological grounds [25, 26]. To gain a comprehensive understanding of each PHI scale-up we retained, we contacted study authors and conducted further literature searches based on citations, name of the PHI, and authors’ names. To ensure a rich understanding of the scale-up process, we searched and gathered as much information as possible regarding factors that may impact the scale-up process, including descriptions of the PHI, its context, and stakeholders involved. To reduce the risk of publication bias, we included peer-reviewed and grey literature.

### Data extraction and analysis

All retained literature was reviewed using a standardized data extraction form designed to capture potential factors and actions relevant to scale-up success (see Additional file 3). We used NVivo10™ to store and code our extracted data and team notes. Themes (i.e., patterns across the data) were explored and identified through the iterative process of coding, categorizing, and conceptualizing [33]. As new data were extracted, they were compared to existing data. Scale-up success was determined based on the authors’ benchmarks; when not explicitly stated, we made a judgment of the success by examining the authors’ descriptions and conclusions.

With the aid of theories of diffusion of innovation, complex adaptive systems, and organizational readiness for change, we posed critical questions for each PHI scale-up attempt (see Additional file 4). The purpose of these questions was to deconstruct the data and assumptions behind our analyses, and draw new insights and interpretations [34]. The guiding theories helped provide meaning, logic, and organization to the data, and provided explanations to the mechanism and significance of specific actions involved with scale-up success [35]. We also used mind mapping and concept mapping techniques to aid our conceptualization of scale-up and the identification of themes across the data set [36, 37]. Mind maps encourage spontaneous exploration with the aim of creating associations between concepts and ideas – depicting new non-linear ways of understanding a topic. Building upon mind maps, concept mapping may advance one’s understanding by outlining the relationships between ideas and identifying the potential impact and influences they may have on one another [22, 23]. Using hand-drawn images and NVivo 10™ visual tools, we created numerous maps and figures to help examine the different depths, categories, and dimensions of scale-up, and relationships between complex concepts and themes (see Additional file 5 for illustrative examples) [38].

**Figure 1:**
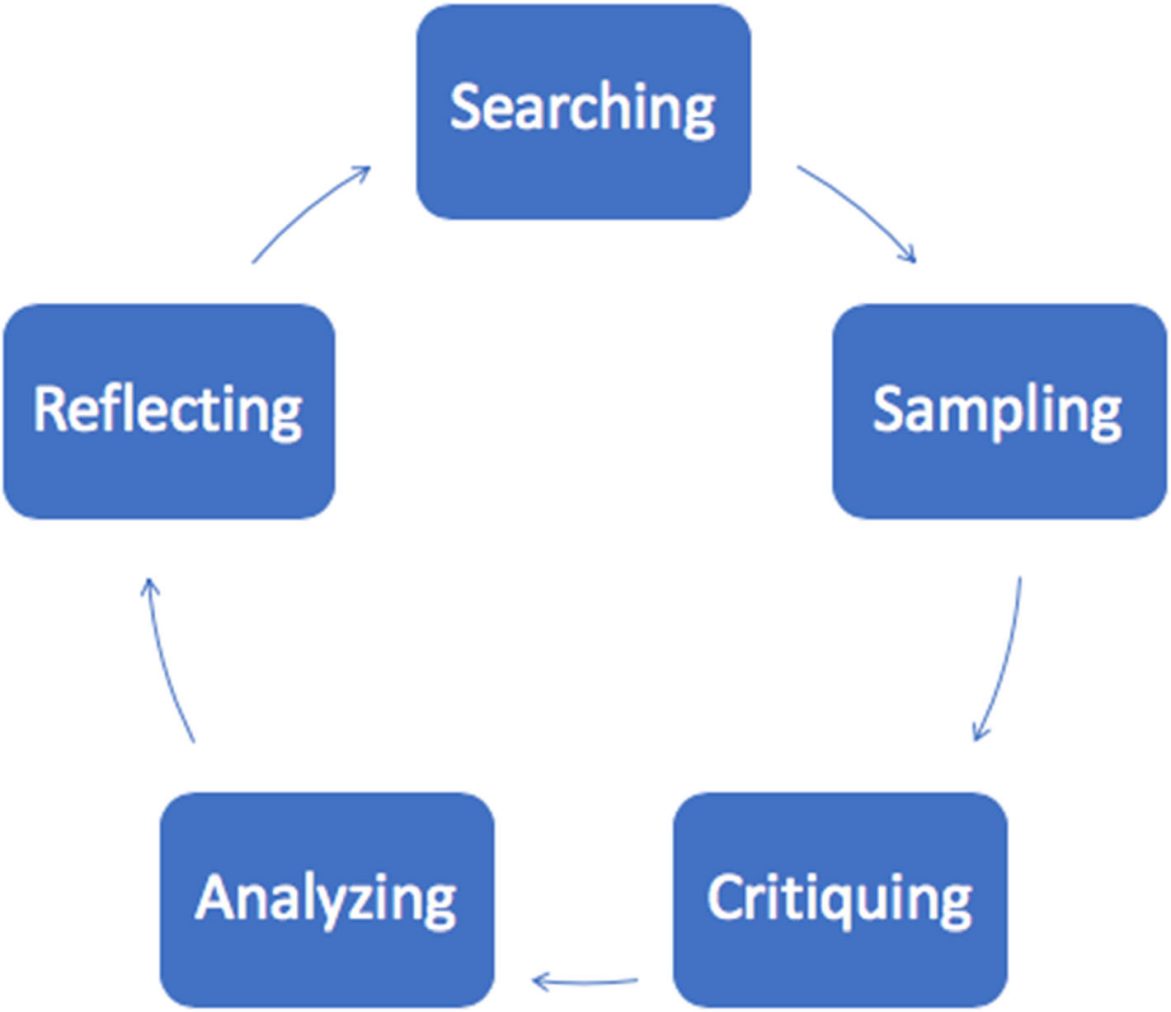
Fig. 1 Critical interpretive sysnthesis (CIS) iterative approach

Searching and sampling was conducted iteratively with data extraction, analysis, and reflection (see Fig. 1 for the iterative approach of a CIS), and continued until saturation was reached [39, 40]. To enhance the rigour of our synthesis, we embedded several strategies, such as sampling adequacy (see Table 2). Sampling adequacy refers to obtaining an appropriate sample for the research topic. To ensure an appropriate sample was obtained, we sought data saturation (i.e., prominent, recurring patterns across multiple studies that are relevant to the research questions) [41, 42]. Prominent qualitative researchers warn data saturation (i.e., informational redundancy) may be prematurely reached when one’s sampling frame is too narrow or skewed [33, 43]. To prevent falsely achieving saturation, we purposely gathered a heterogeneous literature sample of rich, descriptive, scale-up experiences, which includes attempts to gather a comprehensive understanding of the scale-up process [44]. Our retained literature varied on many factors, for example, across different disciplines, health foci, using diverse scale-up techniques. Other rigour strategies we employed in our synthesis include triangulation (i.e., cross-referencing) and active analytic stance (i.e., collecting and analyzing data concurrently) [26, 44].

**Table 2:** Rigour techniques for qualitative research

Strategy for rigour	Description	How it was achieved in this study
Sampling adequacy	This refers to obtaining an appropriate sample for the research topic	To ensure an appropriate sample was obtained, we sought data saturation (i.e., prominent, recurring patters across multiple studies that are relevant to the research question). To avoid falsely achieving saturation, we purposely gathered a heterogeneous sample of the literature that was conceptually rich and depicted the scale-up process in-depth. To increase our likelihood of correctly achieving saturation; we aimed to be inclusive of all potentially relevant studies that may inform this critical synthesis.
Triangulation	This refers to cross-comparing multiple sources to verify the content within, and aid in developing a rich understanding	We retrieved all available literature relevant to the scale-up process for each Population Health Intervention, including empirical studies, reports, commentaries, webpages, and presentations from peer-reviewed and grey literature.
Active analytic stance	This refers to collecting and analyzing data concurrently, to help the researcher better identify what is known and what needs to be examined further	As we began data collection and our concurrent analyses, we became more familiar with the data and gained greater insight into the meaning of the data. We compared new data with existing data to examine whether they supported or refuted our existing themes - this approach helped inform our future data collection. As this process progressed, we noticed that themes became repetitive, providing evidence of verification and completeness of the results.

## Results

We retained 20 PHI scale-up experiences described in 77 documents (47 peer-reviewed; 30 grey literature), published between 1995 and 2013 (Additional file 6). The scale-up experiences occurred in 17 countries, mainly low- (*n* = 5) and middle-income countries (*n* = 9; Additional file 7). These PHIs addressed diverse health issues, using various scale-up approaches. Sixteen PHIs were successful in implementing scale-up, three had mixed results, and one was unsuccessful. Sustainment success occurred in 12 PHIs; four had mixed results; and for the remainder, sustainability was unknown or not applicable.

### Synthesis: foundations of the scale-up readiness assessment framework

Based on our critical synthesis of our 20 real-life scale-up experiences, we identified 11 key actions involved in the successful scale-up of a PHI; as described below these actions were organized over three progressive phases (i.e., Groundwork preparation phase; Implementing scale-up phase; and Sustained the scaled-up PHI phase). In addition, we found that all scale-up attempts encompass four key components: i) the PHI, ii) context/environment, iii) capacity, and iv) stakeholders. Together, these actions and components constitute the foundational content of the Scale-up Readiness Assessment Framework. Below, we describe the phases and actions of the scale-up process, followed by the key scale-up components. To sufficiently describe the richness of the actions, below we provide detail on only one action per phase; however, readers may refer to Nguyen [24] for a full description of all the key actions involved in a successful scale-up of a PHI.

### Scale-up phases and actions

Our findings include key phases and actions that are important and commonly found in a successful scale-up of a PHI, which spans the entire scale-up process. Table 3 provides a brief description of the key phases and its respective actions, showcasing the pathway towards a successful scale-up of a PHI. For ease of presentation the actions in Table 3 are listed in a linear format, however it should be noted that some actions may be conducted in tandem with other actions, while others may be conducted in an iterative or cyclic manner. In other words, our findings revealed the scale-up process does not unfold in a linear fashion. Due to the wide variation encountered in the literature, we did not identify a universal pathway that led to scale-up success in all cases; thus, we refrained from numbering the key actions in the scale-up process and instead offered a general sequence of events that was observed in the literature.

**Table 3:** Key phases and actions in the pathway to successfully scaling-up a PHI

Phase	Action	Description
Phase 1: Groundwork preparation		Groundwork phase includes 5 key actions and refers to prepatory actions conducted prior to implementing scale-up. The primary purpose of Phase 1 is three-fold: i) create a rigorous and systematic scale-up plan; ii) provide sufficient information for decision-makers to make an informed decision about whether to implement scale-up; and iii) develop a strong foundation for subsequent scale-up phases.
Stimulating consideration to scaling-up a PHI		To begin the scale-up process, one or more stimulus is required to incites dialogue or action regarding interest to increase the impact of an existing PHI.
Maintaining existing, and building new, stakeholder engagement and buy-in		Human resources are essential to scale-up, and therefore stakeholders must be engaged early and continuously throughout the process. Stakeholders provide the resources, skills, expertise, management, and coordination required to carry out the long and complex scale-up process. Four broad groups of stakeholders were identified: implementers, receivers/adopters, supporters, and opponents of scale-up.
Conducting/Reviewing assessments		To guide scale-up planning and execution, there are several essential pieces of information that need to be gathered. For example, assessments and data gathered during monitoring and evaluations are pivotal in guiding and informing scale up planning and decisions, such as whether to scale-up, what to scale-up, how to scale-up, where to scale-up, and when to scale-up.
Developing/Retaining/Refining/Modifying resources and stakeholder groups		Throughout the prepatory process there will be actions required to develop, retain, refine, and/or modify various components (i.e., PHI, stakeholders, context, & capacity) of the scale-up process. For example, with respect to stakeholders, different people or organizations may need to be engaged due to changing roles and responsibilities, changing priorities, competing interests, etc.
Deciding whether to implement scale-up of an existing PHI		Concluding the preparatory phase of scale-up, a decision will need to be made regarding whether or not to scale-up the PHI. Deliberations are conducted, typically by a committee of key stakeholders, regarding actions to scale-up a PHI. Many factors go into the decision-making process (e.g., evidence of health impacts; stakeholder commitment, cost-effectiveness), and the ranked importance of such factors vary between decision makers.
Phase 2: Implementing scale-up		Implementing Scale-Up Phase includes 4 key actions. Implementation refers to the process of executing scale-up of the PHI; this phase is only conducted if the PHI is strongly being considered for scale-up. The primary purpose of phase 2 is three-fold: i) successfully implement scale-up; ii) prepare to sustain the scaled-up PHI; and iii) decide how long to sustain the scaled-up PHI.
Continuing / Modifying actions conducted during Groundwork Phase		This action reflects the iterative and dynamic actions of scale-up, and the need to occasionally continue or build-up previous actions. Many previous actions may either be continuing with or without modifications, for example because the focus shifts towards implementing scale-up, unintended consequences, or lessons learned.
Building / Consolidating capacity for scale-up		Scaling-up requires many different capacities. Sufficient capacity for scale-up is typically accumulated over time, by way of newly acquiring and/or consolidation. There are various capacities required for scale-up (e.g., PHI design, infrastructure, resources, financial, technical).
Rolling out scale-up implementation strategies		Various strategies may be used to implement scale-up of a PHI (e.g., decentralization; integration; replication). Typically, implementation is conducted in a phased or incremental manner over an extended period of time. Occasionally inspections or fines must be enforced to ensure scale-up is being implemented as intended.
Deciding whether to sustain the scaled-up PHI		At some point during the implementation phase, a decision must be made regarding whether the scaled-up PHI should and will be sustained. Sustaining the scaled-up PHI for a longer length of time may not be applicable to all scenarios (e.g., due to the nature of the health issue; availability of resources; changing priorities), and this decision will be unique to the scale-up scenario.
Phase 3: Sustaining the scaled-up PHI		Sustaining the scaled-up PHI phase includes 2 key actions. Sustaining refers to sustaining the effort to maintain the scaled-up PHI and, thereby sustaining the impact of the scaled-up PHI. The primary purpose of Phase 3 is to successfully sustain the scaled-up PHI for the intended period of time.
Continuing / Modifying previous actions to maintain the scaled-up PHI		This action includes an assortment of previous actions undertaken in the two previous phases. Many previous actions may either be continuing with or without modifications depending on the changing circumstances of the scale-up scenario. The focus shifts from implementing scale-up to maintaining the scaled-up PHI, and due to this shift actions are adjusted accordingly.
Adapting / Evolving to changing components		To assist in sustaining the scaled-up PHI for an extended length of time, some may need to adapt or evolve their scaled-up PHI based on changes to the key components of scale-up (i.e., context, stakeholders, & capacity).

### Phase 1: Groundwork preparation

Scaling-up is a long, complex, and iterative process, and this is reflected in the actions we identified. Actions within this first phase of scale-up are essential to building the foundation of the scale-up process, and some will continue and/or be modified throughout the scale-up process in a consistent, iterative, or cyclical fashion throughout subsequent phases; although the action’s purpose may change along the way. For example, at the beginning of the scale-up phases, scale-up practitioners may be focused mainly on engaging buy-in from key stakeholders (i.e., Phase 1: Groundwork Phase action: *Maintaining existing, and building new stakeholder engagement and buy-in*), but as the process progresses the primary focus may shift to maintaining stakeholder buy-in (i.e., Phase 2: Implementing Scale-up action: *Continuing/Modifying actions conducted during the Groundwork Phase*). The continuation of actions, such as the example we just described, is essential to maintain the momentum of the action and/or build upon other actions.

The primary purpose of Phase 1 is three-fold: i) create a rigorous and systematic scale-up plan; ii) provide sufficient information for decision-makers to make an informed decision about whether to implement scale-up; and iii) develop a strong foundation for subsequent scale-up phases. With the exception of the first action of this phase, *Stimulating consideration to scaling-up a PHI*, these actions take place after the PHI is developed, and continue until a decision is made regarding whether or not to scale-up the PHI. Groundwork preparation is the most important and labour intensive of all scale-up phases.

One of five key actions we categorized in the Groundwork Preparation phase is, *Maintaining existing, and building new, stakeholder engagement and buy-in.* We identified four broad groups of stakeholders involved in scale-up: i) implementers; ii) receivers/adopters; iii) supporters; and iv) opponents. For each scale-up attempt, there is a complex web of stakeholders who fulfill various roles and responsibilities, which vary by: i) complexity of the PHI; ii) context; iii) scope of scale-up; and iv) scale-up approach. Typically, diverse and dedicated stakeholder teams (e.g., groups, committees, tasks forces) are needed to facilitate necessary scale-up actions. Early engagement and buy-in of various stakeholders from multiple disciplines/sectors was found to facilitate robust commitment and advocacy for scale-up (PHI #5: Evelia et al., 2008; PHI #6: Fajans et al., 2007). A common strategy to gain and maintain buy-in is regular communication (e.g., meetings, progress reports, consultations, participatory strategies, media; PHI #14: Libamba et al., 2006). Having a diverse group of stakeholders helps broaden and raise awareness of the PHI, build influence (PHI #6: Fajans et al., 2007), and enhance opportunities to obtain and retain key resources and supports (PHI #1: Binagwaho et al., 2012). This was particularly true when buy-in was attained from influential stakeholders who were perceived to have credibility and/or power (e.g., gatekeepers, World Health Organization, Bill and Belinda Gates Foundation; PHI #2: Chowdhury et al., 2006). As the scale-up process progresses, more stakeholders are required to fulfill new/growing responsibilities (PHI #5: Evelia et al., 2008; PHI #8: Friedland et al., 2007). As more stakeholders join the scale-up process, a sophisticated strategy to coordinate and manage the various roles and responsibilities is viewed as an asset (PHI #13: Kaufman et al., 2007; PHI #17: Renju et al., 2010b).

If the decision during the final action of the Groundwork phase is to pursue scale-up, PHI stakeholders may continue to the next scale-up phase.

### Phase 2: Implementing scale-up

Implementation refers to the process of delivering scale-up of the PHI; this phase is only conducted if the PHI is strongly being considered for scale-up. The primary purpose of Phase 2 is three-fold: i) successfully implement scale-up; ii) prepare to sustain the scaled-up PHI; and iii) decide how long to sustain the scaled-up PHI.

One of four key actions identified in Phase 2 is *Building & consolidating capacity for scale-up.* Scale-up capacity was observed in several areas, including the PHI design, infrastructure, resources, and technical knowledge and skills. Often the PHI had to be customized to the scale-up site (PHI #2: Chowdhury et al., 2006; PHI #11: Hoek et al., 2010). To ensure sites had the proper infrastructure and scale-up capacity, they were typically reviewed, screened, and examined (e.g., field visits, stakeholder meetings). During early stages when scale-up challenges arose (e.g., lack of necessary supplies/space), stakeholders tried to address them before rolling out scale-up of the PHI (PHI #10: Gonzales et al., 1998). Building technical capacity ordinarily required training and flexibility to adapt to local conditions – especially in resource-poor settings where skilled workers were limited, over-burdened with responsibilities, and time-constrained (PHI #5: Evelia et al., 2008; PHI #12: Huicho et al., 2005a). Moreover, supervisory check-ups and booster training sessions were commonly required to help solidify lessons learned and allow trainees an opportunity to clarify areas of confusion (PHI #14: Libamba et al., 2006). This was particularly important in countries where workers were inundated by various training programs from external organizations that also aimed to build capacity (PHI #9: Gloyd et al., 2007). A related issue was identifying who would benefit most from capacity training, as training and resources were limited. Finding the right balance between efficiency, training quality, and practical constraints was a constant challenge. Supervision was also helpful to manage and ensure necessary scale-up resources (e.g., finances, technologies, medical supplies, tools, training modules) were available (PHI #14: Libamba et al., 2006). Securing procurement and an uninterrupted supply of necessary resources aided in scale-up success, as PHI workers were then able to carry out and maintain the scaled-up PHI as intended.

### Phase 3: Sustaining the scaled-up PHI

The final phase of the scale-up process includes sustaining the scaled-up PHI, after (parts or all phases) of scale-up implementation of the PHI was successfully completed. Phase 3 has two key actions, with a primary purpose to successfully sustain the scaled-up PHI for the intended period of time, in a responsible and appropriate manner. The primary purpose of Phase 3 is to successfully sustain the scaled-up PHI for the intended period of time.

One of two actions in Phase 3 is *Adapting/Evolving to changing components.* Some PHIs must adapt or evolve, depending on the level of influence the key components (described next) have on the scaled-up PHI. Adaptation refers to subtle adjustments complementing changes in the key components (PHI #15, Njau et al., 2009). For example, in the implementation of an injury prevention law, Passmore et al. (PHI #16: 2010) reported, “[s] hortly after the introduction of the legislation, several loopholes that had the potential to reduce its effectiveness were identified” (p. 783) and later corrected. Evolution refers to transformation of the PHI to a point where it is no longer recognizable. Examples of evolution include a PHI that broadened its health focus following scale-up (PHI #15: Njau et al., 2009); changed its name to reflect major changes within the PHI (PHI #10: Pan American Health Organization, 2008); and a circumstance where only a portion of the scaled-up PHI was maintained (PHI #12: Huicho et al., 2005a).

### Scale-up components

Despite the variation found between each scale-up experience, we identified four key components that were present in every scale-up attempt: i) the PHI, ii) context/environment, iii) capacity, and iv) stakeholders (Table 4). Together, these components worked as a unified, complex system and were connected, interdependent and constantly changing, evolving, and had an influence on the scale-up process. Understanding these components provided insight into the variation that existed between each scale-up experience and provided some explanation of the scale-up process and outcome.

**Table 4:** Key scale-up components

Scale-up component	Description
Population health intervention (PHI)	A discrete set of actions that impact a number of people by attending to underlying conditions (i.e., social determinant of health) of risk, thereby improving population health and reducing health inequities
Context	The social, cultural, physical, political, and organizational settings within which a PHI is implemented and sustained. Resources refers to the supplies required for the scale-up process, including time, finances, tools/technology, documents, and facilities
Capacity	One’s current and potential ability to carry out the scale-up process. Potential capacity may be measured by one’s ability to obtain the necessary resources (e.g., materials, supplies), skills and competencies, and support for scale-up, as well as high levels of commitment, patience, coordination, and drive for follow-through
Stakeholders	People and organizations that are connected to the scale-up process; this includes the implementers of the intervention (e.g., designers of PHI, collaborators), adopters of the intervention (e.g., organizations who will take the scaled up PHI), receivers of the intervention (e.g., target population), supporters of the intervention (e.g., funders, partners), and opponents of scale-up (e.g., groups with conflicting interest)

## Discussion

This synthesis was driven by variable scale-up success experienced in practice and by gaps and concerns reported in the scale-up literature; specifically, simplistic conceptualizations of the scale-up process [3], lack of focus on sustainability [17], and scarcity of theory [15, 16]. Since our literature search, new scale-up literatures continue to add to our understanding of the scale-up process. Our work builds upon the extant literature in several meaningful ways.

First, our synthesis diverges from previous simplistic conceptualization of scale-up, by drawing from real-life scale-up experiences, highlighting the complexity of the scale-up processes, and the various paths that may be taken to scale-up success. Our analysis revealed the process to successfully scaling-up a PHI to be unique, iterative, and nuanced. In the literature no two scale-up experiences were the same, and to help illustrate this point, we identified a general sequence of actions that were common to many successful scale-up experiences. We avoided numbering our scale-up actions, to signify there is no prescriptive, universal approach to successful scale-up, and actions may occur sequentially, iteratively, and in tandem with other actions. To be able to assess the scale-up team’s readiness for scale-up, it is important the scale-up team is aware of these important scale-up characteristics, so they may be adequately prepared and be encouraged to make thoughtful decisions based on their needs and context.

Second, to help advance our conceptualization of scale-up, we built upon previous frameworks that have incorporated theories such as diffusion of innovations (e.g., [5]) and complex adaptive systems (e.g., [3, 19]), by also including organizational readiness for change as one of our three guiding theories [20]. To our knowledge, only two scale-up studies have focused on readiness. The WHO [23] developed a landscape analysis on countries’ readiness of nutrition interventions through interviews and workshops, and Moran and colleagues [21] examined scale-up readiness for neonatal interventions by developing a set of benchmark indicators. Due to the complexities that exist both within a scale-up process, and the variation that exist between the different PHI scale-up experiences in the literature, we suggest that readiness for a PHI may be better assessed on a case by case basis, by reviewing the key actions involved in scale up and comparing it to one’s own circumstance.

The organizational readiness for change theory recommends understanding all key actions needed for scale-up, from conceptualization to sustainment, to help improve the likelihood for success. When examining the literature, the change theory helped explain why the Groundwork Phase is foundational to the overall scale-up success. Actions in the Groundwork Phase have an impact on all subsequent actions in the scale-up process, including its overall success. Similar to the work of ExpandNET and the World Health Organization [9], we found it was essential to prepare for sustainability at the forefront of the scale-up process. By not understanding what is required for scale-up at the beginning, one will not know what to prepare or how to plan, thus negatively impacting the likelihood for scale-up success. Our paper adds to the literature by supporting past studies that have emphasized the importance of readiness for successful scale-up.

Finally, there is still a paucity of scale-up literature that focus specifically on sustaining scale-up [17]. Our work highlighted some actions that were used for sustaining a scaled-up PHI in particular. The intended length of time to sustain a scaled-up PHI may vary, and the decision will be dependent on the unique scaling-up context. There is still much more that can be learned about sustaining a scaled-up intervention, but we hope that others may build upon our findings and that future research may include further details of the scale-up process following implementation, so that we may better learn from these collective experiences.

### Limitations

One main limitation was the relatively limited number of rich, descriptive studies available. There were many studies that discussed the scale-up of a PHI, but most had a quantitative focus and were more concerned with scale-up outcomes (e.g., cost-effectiveness, health impacts) than documenting the process itself. Of the studies that discussed the scale-up process, many lacked depth, and even fewer included details regarding the sustainment of scale-up -- thus resulting in an imbalanced examination of the scale-up process. Additionally, the CIS method cannot produce a synthesis that in of itself is externally validated. While this synthesis provides the foundations for future research and practice efforts, this Framework’s content validity, as well as its utility for its intended user’s needs further assessment, which was part of the larger study and will be reported in a future paper.

## Conclusion

Overall, our work advances the scale-up literature in several ways. First, our framework is theoretically coherent with explanatory power from three robust theories (i.e., diffusion of innovation, complex adaptive systems, organizational readiness for change). Second, we provide another perspective to assessing scale-up readiness, that focuses on one’s own circumstance in relation to key phases and actions of a scale-up process. Third, to best capture the complexities of successfully scaling-up a PHI and reduce the likelihood of prematurely drawing a conclusion, we deliberately gathered a heterogenous sample of PHIs scale-up experiences. Through our analysis of diverse PHI scale-up attempts located from various sources, we identified key aspects (phases and actions) involved in the process of successfully scaling-up a PHI. With a more detailed understanding of the complex and nuanced actions involved in the pathways to successful scale-up, PHI stakeholders may better examine the necessary actions and capacities against their own, and make an informed judgment regarding whether and how to proceed with scale-up. More broadly, the foundations of our Scale-up Readiness Assessment Framework contribute to strengthening the science and practice of scale-up for the unique circumstances of PHIs.

## Supplementary information

**Additional file 1:** Search terms.

**Additional file 2:** Websites searched.

**Additional file 3:** Standardized data extraction form.

**Additional file 4:** Critical questionsposed during analysis.

**Additional file 5:** Maps and visualizations created during analysis.

**Additional file 6:** References of the retained literature. Abibliographical list of all literature retained for the critical interpretive synthesis.

**Additional file 7:** Summary of the retained literature ( n = 77).

## Data Availability

All data gathered and analyzed for this review are included in this published article (and its Additional files).

## Abbreviations

CAS: Complex adaptive systems
CIS: Critical interpretive synthesis
PHI: Population health intervention
