# Correction to: Developing a framework to inform scale-up success for population health interventions: a critical interpretive synthesis of the literature

**DOI:** 10.1186/s41256-020-00157-0

**Published:** 2020-06-05

**Authors:** Duyen Thi Kim Nguyen, Lindsay McLaren, Nelly D. Oelke, Lynn McIntyre

**Affiliations:** 1 Department of Economics, Faculty of Business, University of New Brunswick, 100 Tucker Park Road, P.O. Box 5050, Saint John, New Brunswick E2L 4 L5 Canada; 2 School of Epidemiology and Public Health, Faculty of Medicine, University of Ottawa, 600 Peter Morand Crescent, Ottawa, Ontario K1G 5Z3 Canada; 3 New Brunswick Health Research Foundation, 10 Knowledge Park Drive, Fredericton, New Brunswick E3C 2 M7 Canada; 4 Department of Community Health Sciences, Cumming School of Medicine, University of Calgary, TRW3, 3280 Hospital Dr. NW, Calgary, Alberta T2N 4Z6 Canada; 5 O’Brien Institute for Public Health, University of Calgary, TRW3, 3280 Hospital Dr. NW, Calgary, Alberta T2N 4Z6 Canada; 6 School of Nursing, Faculty of Health and Social Development, University of British Columbia, 3333 University Way, Kelowna, British Columbia V1V 1 V7 Canada

**Correction to: Global Health Res Policy (2020) 5:18**

**https://doi.org/10.1186/s41256-020-00141-8**

Following the publication of the original article [1], it was noted that due to a typesetting error the Table 3 layout was incorrect. The correct table is given below.

**Table 3:** Key phases and actions in the pathway to successfully scaling-up a PHI

Phase	Action	Description
Phase 1: Groundwork Preparation		Groundwork phase includes 5 key actions and refers to preparatory actions conducted prior to implementing scale-up. The primary purpose of Phase 1 is three-fold: i) create a rigorous and systematic scale-up plan; ii) provide sufficient information for decision-makers to make an informed decision about whether to implement scale-up; and iii) develop a strong foundation for subsequent scale-up phases.
	Stimulating consideration to scaling-up a PHI	To begin the scale-up process, one or more stimulus is required to incites dialogue or action regarding interest to increase the impact of an existing PHI.
	Maintaining existing, and building new, stakeholder engagement and buy-in	Human resources are essential to scale-up, and therefore stakeholders must be engaged early and continuously throughout the process. Stakeholders provide the resources, skills, expertise, management, and coordination required to carry out the long and complex scale-up process. Four broad groups of stakeholders were identified: implementers, receivers/adopters, supporters, and opponents of scale-up.
	Conducting/Reviewing assessments	To guide scale-up planning and execution, there are several essential pieces of information that need to be gathered. For example, assessments and data gathered during monitoring and evaluations are pivotal in guiding and informing scale up planning and decisions, such as whether to scale-up, what to scale-up, how to scale-up, where to scale-up, and when to scale-up.
	Developing/Retaining/Refining/Modifying resources and stakeholder groups	Throughout the preparatory process there will be actions required to develop, retain, refine, and/or modify various components (i.e., PHI, stakeholders, context, & capacity) of the scale-up process. For example, with respect to stakeholders, different people or organizations may need to be engaged due to changing roles and responsibilities, changing priorities, competing interests, etc.
	Deciding whether to implement scale-up of an existing PHI	Concluding the preparatory phase of scale-up, a decision will need to be made regarding whether or not to scale-up the PHI. Deliberations are conducted, typically by a committee of key stakeholders, regarding actions to scale-up a PHI. Many factors go into the decision-making process (e.g., evidence of health impacts; stakeholder commitment, cost-effectiveness), and the ranked importance of such factors vary between decision makers.
Phase 2: Implementing Scale-Up		Implementing Scale-Up Phase includes 4 key actions. Implementation refers to the process of executing scale-up of the PHI; this phase is only conducted if the PHI is strongly being considered for scale-up. The primary purpose of phase 2 is three-fold: i) successfully implement scale-up; ii) prepare to sustain the scaled-up PHI; and iii) decide how long to sustain the scaled-up PHI.
	Continuing / Modifying actions conducted during Ground-work Phase	This action reflects the iterative and dynamic actions of scale-up, and the need to occasionally continue or build-up previous actions. Many previous actions may either be continuing with or without modifications, for example because the focus shifts towards implementing scale-up, unintended consequences, or lessons learned.
	Building / Consolidating capacity for scale-up	Scaling-up requires many different capacities. Sufficient capacity for scale-up is typically accumulated over time, by way of newly acquiring and/or consolidation. There are various capacities required for scale-up (e.g., PHI design, infrastructure, resources, financial, technical).
	Rolling out scale-up implementation strategies	Various strategies may be used to implement scale-up of a PHI (e.g., decentralization; integration; replication). Typically, implementation is conducted in a phased or incremental manner over an extended period of time. Occasionally inspections or fines must be enforced to ensure scale-up is being implemented as intended.
	Deciding whether to sustain the scaled-up PHI	At some point during the implementation phase, a decision must be made regarding whether the scaled-up PHI should and will be sustained. Sustaining the scaled-up PHI for a longer length of time may not be applicable to all scenarios (e.g., due to the nature of the health issue; availability of resources; changing priorities), and this decision will be unique to the scale-up scenario.
Phase 3: Sustaining the Scaled-Up PHI		Sustaining the scaled-up PHI phase includes 2 key actions. Sustaining refers to sustaining the effort to maintain the scaled-up PHI and, thereby sustaining the impact of the scaled-up PHI. The primary purpose of Phase 3 is to successfully sustain the scaled-up PHI for the intended period of time.
	Continuing / Modifying previous actions to maintain the scaled-up PHI	This action includes an assortment of previous actions undertaken in the two previous phases. Many previous actions may either be continuing with or without modifications depending on the changing circumstances of the scale-up scenario. The focus shifts from implementing scale-up to maintaining the scaled-up PHI, and due to this shift actions are adjusted accordingly.
	Adapting / Evolving to changing components	To assist in sustaining the scaled-up PHI for an extended length of time, some may need to adapt or evolve their scaled-up PHI based on changes to the key components of scale-up (i.e., context, stakeholders, & capacity).

The original article has been corrected.
