# Supplementary material for: Developing a framework to inform scale-up success for population health interventions: a critical interpretive synthesis of the literature
Source: Glob Health Res Policy. 2020 Apr 29;5:18. doi: 10.1186/s41256-020-00141-8 (PMC7189598; doi:10.1186/s41256-020-00141-8)
Supplement: Supplementary file 1 — Additional file 1: Search terms. [file 41256_2020_141_MOESM1_ESM.docx]

**Additional file 1:**

**Search terms**

Keyword and phrase searches within titles and abstracts were conducted for electronic databases

Terms and their variants used to capture scaling-up included: scale-up, scaling-up, spread, replication, integration, universal coverage, increasing impact, increasing effectiveness, improving impact, expanding, and implementation. Terms and their variants used to capture a PHI included: population health intervention, health intervention, health program, health policy, health strategy, public health, health promotion, global health, international health, prevention, and innovatio
