# Supplementary material for: Developing a framework to inform scale-up success for population health interventions: a critical interpretive synthesis of the literature
Source: Glob Health Res Policy. 2020 Apr 29;5:18. doi: 10.1186/s41256-020-00141-8 (PMC7189598; doi:10.1186/s41256-020-00141-8)
Supplement: Supplementary file 2 — Additional file 2: Websites searched. [file 41256_2020_141_MOESM2_ESM.docx]

**Additional file 2:**

**List of relevant websites searched**

| 1. National Implementation Research Network: <http://nirn.fpg.unc.edu/resource-search> 2. Public Health Agency of Canada: <http://www.phac-aspc.gc.ca/publications-eng.php> 3. International Initiative for Impact Evaluation: <http://www.3ieimpact.org> 4. Globalization and Health Equity: <http://www.globalhealthequity.ca> 5. Institute for Healthcare Improvement (IHI) Conference to Advance the State of the Science and Practice on Scale-up and Spread of Effective Health Programs: <http://ihiscaleupconference10.blogspot.ca> 6. International Monetary Fund (IMF) Sustainable investment scaling up in low-income countries: <http://www.imf.org/external/np/seminars/eng/2010/spr/lic/> 7. National Center on Scaling Up Effective Schools: <http://www.scalingupcenter.org> 8. Washington, USA Scale-up Conference (2010): website no longer available   -Example of commissioned paper for the coference: <http://webcache.googleusercontent.com/search?q=cache:sp3zNqhlJ-YJ:www.ihi.org/education/Documents/ProgramMaterials/ScaleUpBlog/7a_Commissioned_Paper%25202_Public_Health.doc+&cd=2&hl=en&ct=clnk&gl=ca&client=safari>   1. Bill & Melinda Gates foundation: <https://www.gatesfoundation.org> 2. Wolfensohn Centre for Development at Brookings: <https://www.brookings.edu/center/wolfensohn-center-for-development/> |
| --- |
