# Supplementary material for: Developing a framework to inform scale-up success for population health interventions: a critical interpretive synthesis of the literature
Source: Glob Health Res Policy. 2020 Apr 29;5:18. doi: 10.1186/s41256-020-00141-8 (PMC7189598; doi:10.1186/s41256-020-00141-8)
Supplement: Supplementary file 3 — Additional file 3: Standardized data extraction form. [file 41256_2020_141_MOESM3_ESM.docx]

**Additional file 3:**

**Critical interpretive synthesis data extraction form**

1. Data Collection

- Name of reviewer:
- Date:
- Full Citation:
- Type of paper (peer-reviewed or grey lit):

2. Abbreviated Abstract

- Study purpose:
- Rationale for scale up:
- Method:
- Outcome of scale up attempt (i.e., Provide a brief description of general scale up process & whether it was successful):

3. Descriptives

- Study Location (Human Development Index):
- Social Determinant of Health focus:
- Type of intervention (e.g., discrete, multi-component, paradigmatic (including policy) & Provide description of intervention):
- Type of scale up (e.g., expansion, diversification, political, organizational, etc. & How was scale up described? The strategies used):
- Intended population:

4. Primary information

- Implementation (any information regarding the implementation of scale up, including the process, actors involved, aids, barriers):
- Sustainability (any information regarding the sustainability of scale up, including the process, actors involved, aids, barriers):

5. Secondary information

- Context (e.g. boundaries; political, social, physical environment; program evaluation; theory behind program; did intervention align w/local norms & values; internal & external resources avail):
- Subsequent outcome of scale up attempt (e.g., Intervention impact; Program evaluation, Unexpected events; Ethical dilemmas/impact, Lessons learned; Did the intervention replace another intervention - if so, what was the impact;):

6. Reflections

- Reflections (e.g., impression of the paper, how it connects with previous studies, etc.):
