# Supplementary material for: Developing a framework to inform scale-up success for population health interventions: a critical interpretive synthesis of the literature
Source: Glob Health Res Policy. 2020 Apr 29;5:18. doi: 10.1186/s41256-020-00141-8 (PMC7189598; doi:10.1186/s41256-020-00141-8)
Supplement: Supplementary file 4 — Additional file 4: Critical questionsposed during analysis. [file 41256_2020_141_MOESM4_ESM.docx]

**Additional file 4:**

**Critical questions regarding each scale-up attempt of a population health intervention**

| Questions |
| --- |
| 1. How does the PHI complexity affect the scale-up process and outcome? |
| 1. How does the PHI context affect the scale-up process and outcome? |
| 1. Do some factors have differing impacts on the scale-up process? If so, why or under what context does this occur? |
| 1. What can be learned when applying the lens of diffusion of innovation, complex adaptive systems, and organizational readiness for change - both individually and collectively? |
| 1. Across these different PHIs, what are some common threads to successful scale-up attempts? |
