# Supplementary material for: Developing a framework to inform scale-up success for population health interventions: a critical interpretive synthesis of the literature
Source: Glob Health Res Policy. 2020 Apr 29;5:18. doi: 10.1186/s41256-020-00141-8 (PMC7189598; doi:10.1186/s41256-020-00141-8)

**Additional file 5:**

**Concept maps and mind maps of the scale up process**

Mind mapping example**
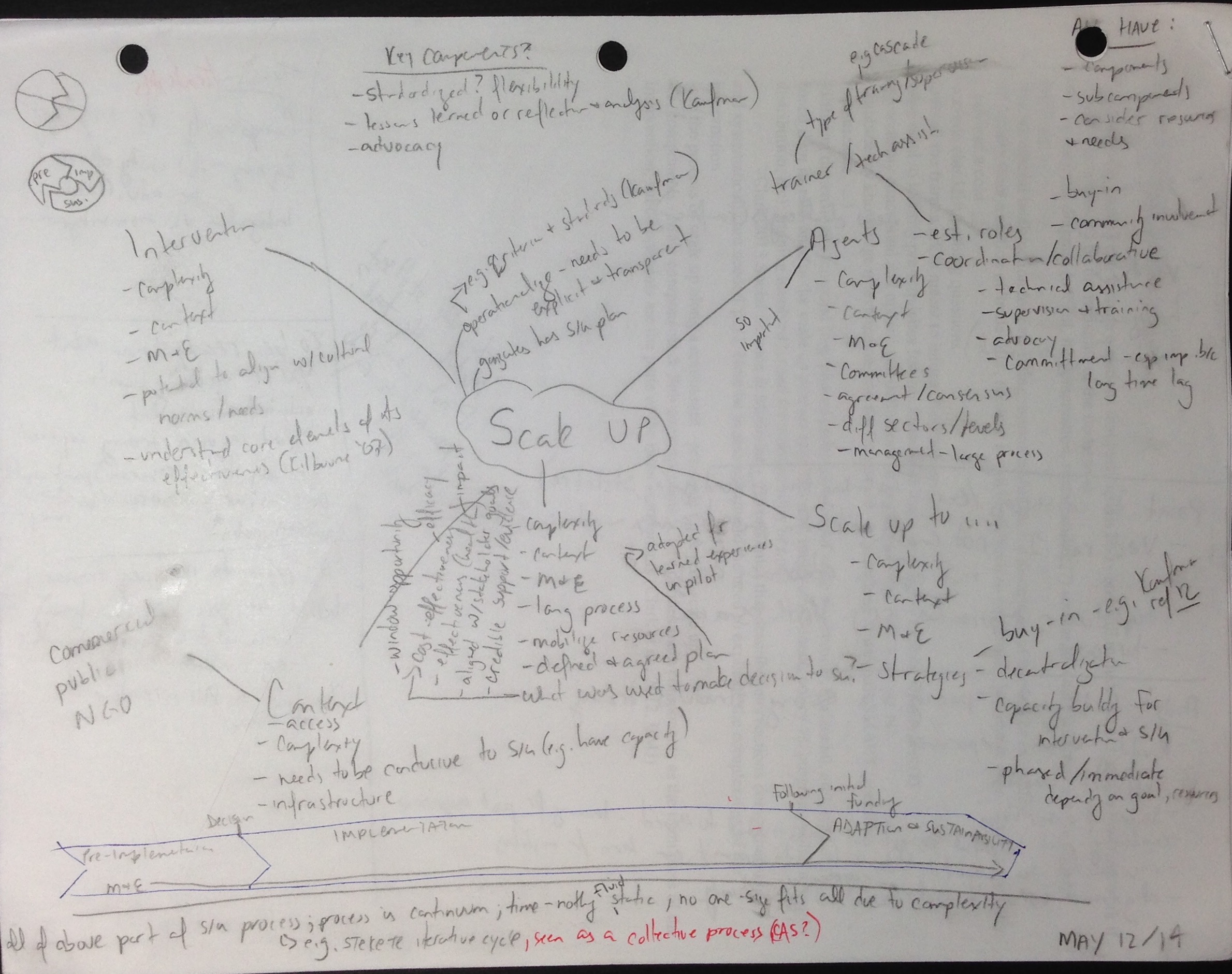
**

Mind mapping example **
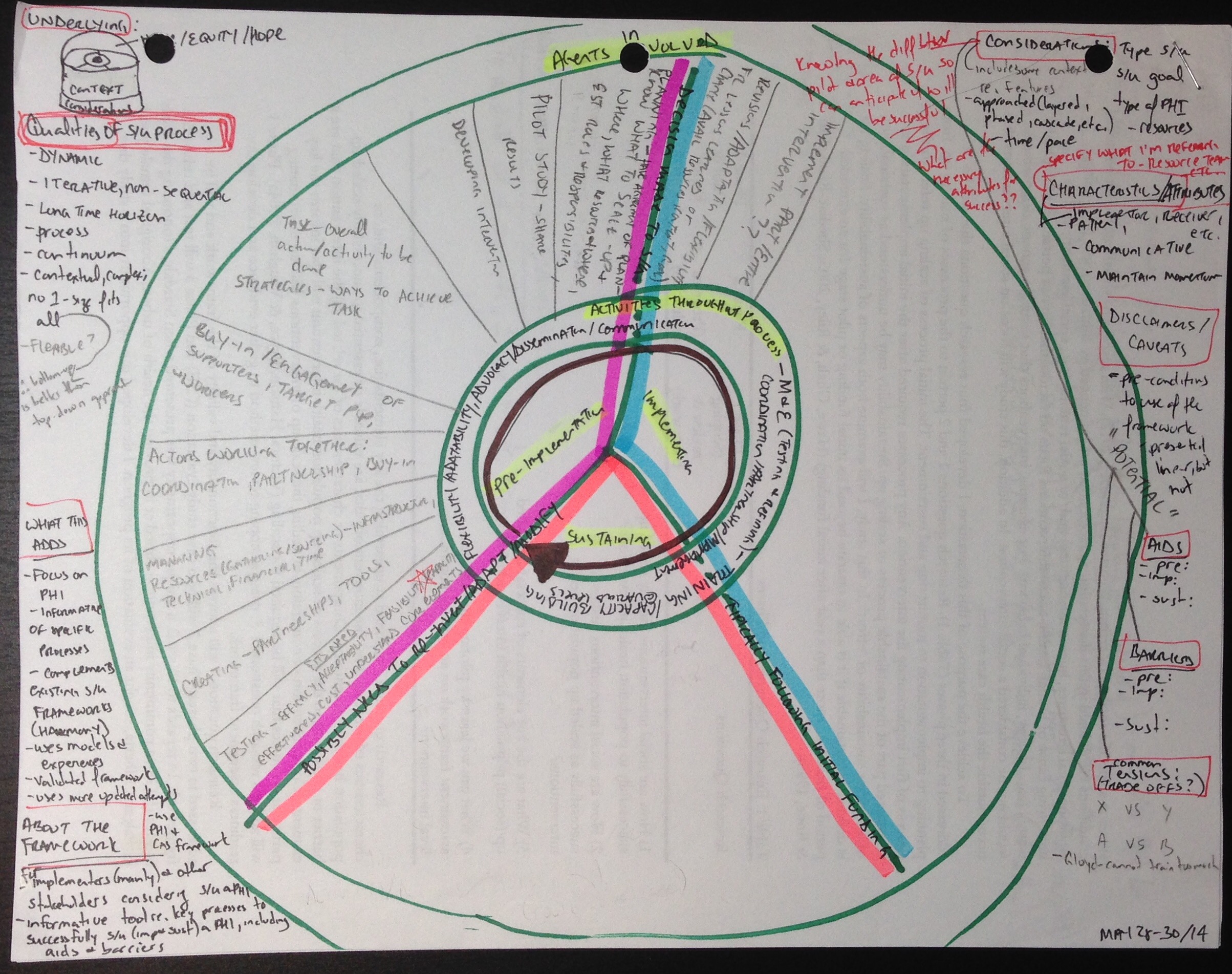
**

Concept mapping example**
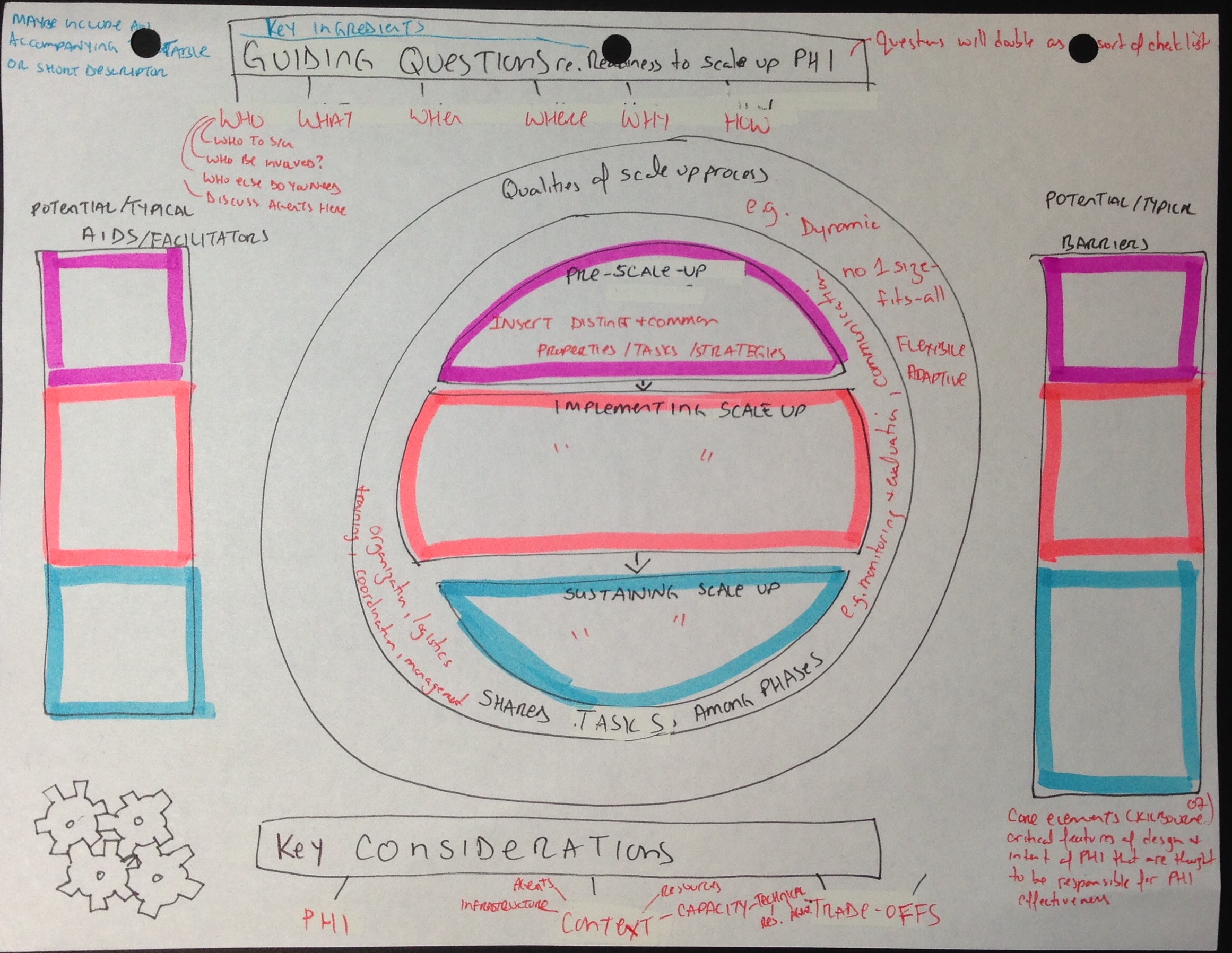
**

NVivo Tree Maps

Nodes compared by number of items coded


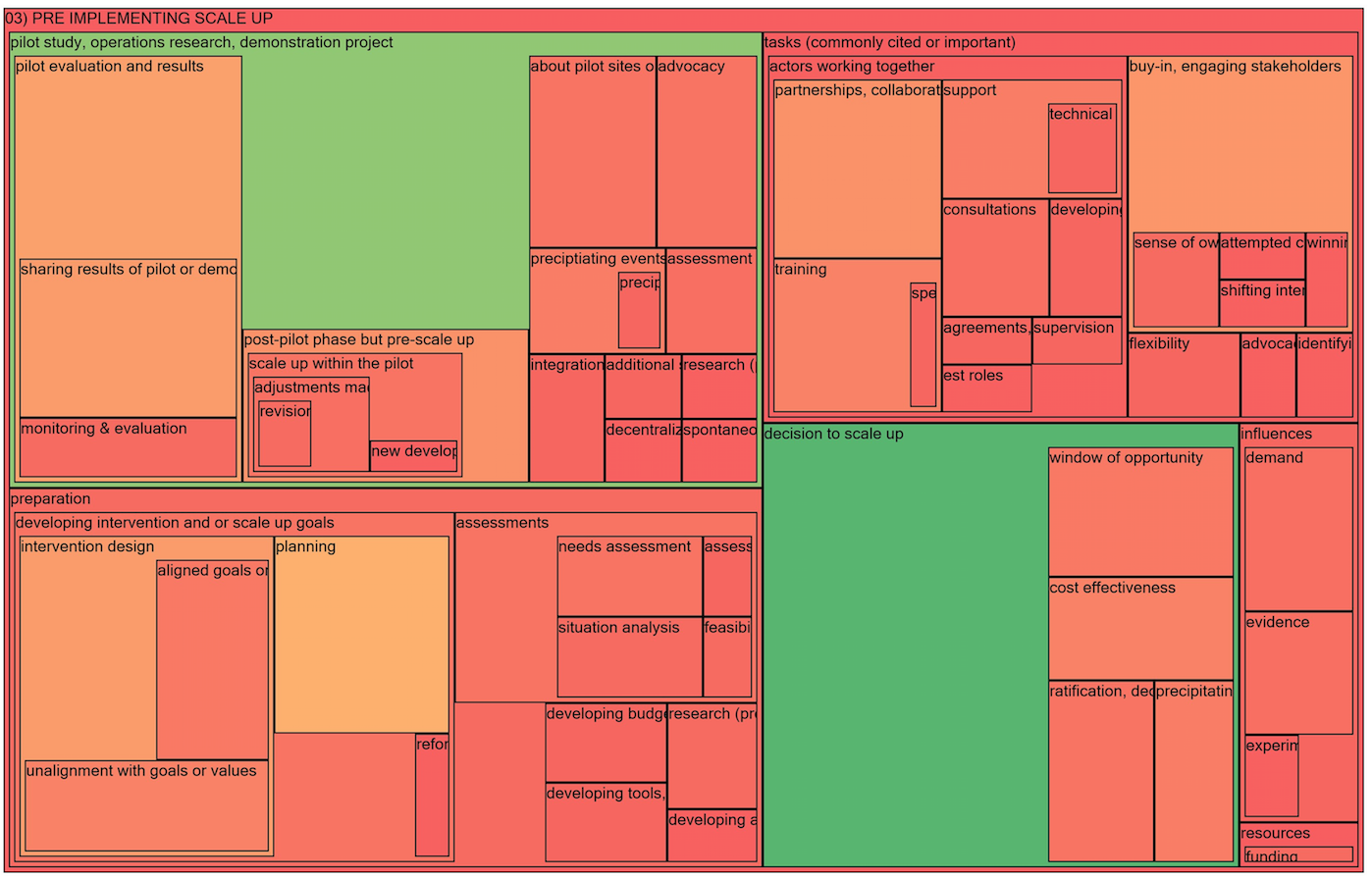


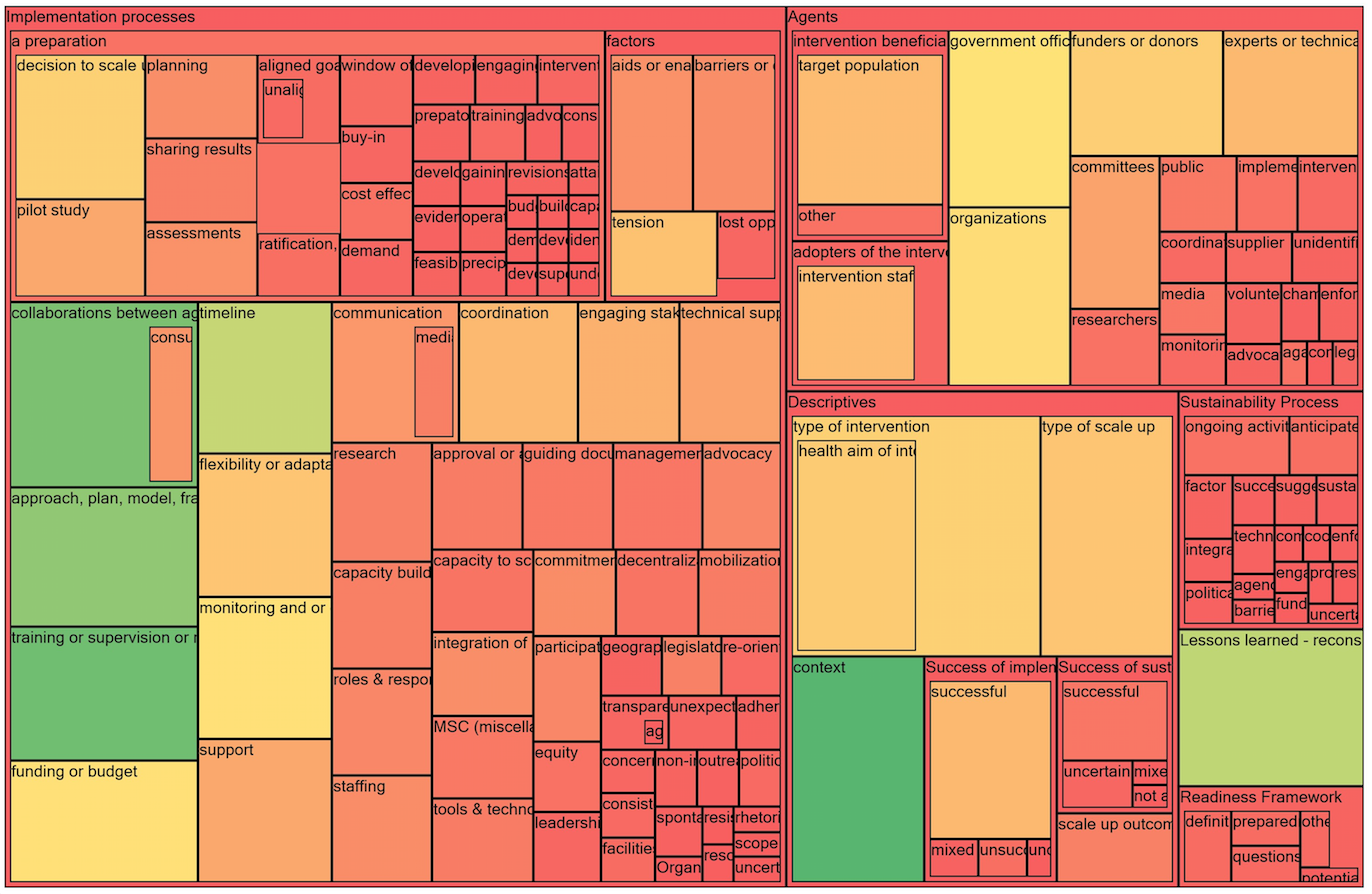


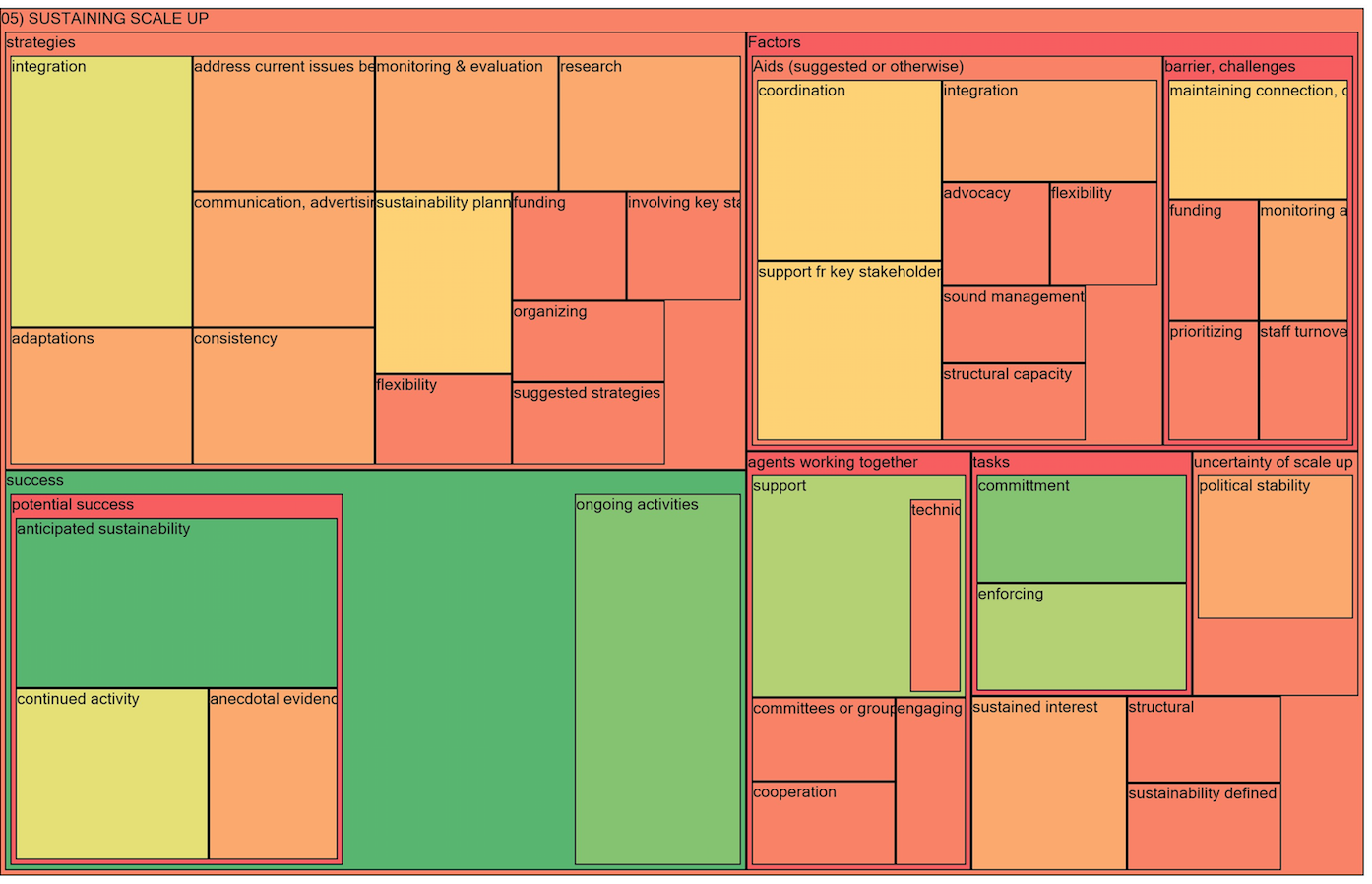

Supplement: Supplementary file 5 — Additional file 5: Maps and visualizations created during analysis. [file 41256_2020_141_MOESM5_ESM.docx]
