# Supplementary material for: Developing a framework to inform scale-up success for population health interventions: a critical interpretive synthesis of the literature
Source: Glob Health Res Policy. 2020 Apr 29;5:18. doi: 10.1186/s41256-020-00141-8 (PMC7189598; doi:10.1186/s41256-020-00141-8)
Supplement: Supplementary file 6 — Additional file 6: References of the retained literature. Abibliographical list of all literature retained for the critical interpretive synthesis. [file 41256_2020_141_MOESM6_ESM.docx]

**Additional file 6:**

**References of retained studies**

Askew I, Chege J, Njue C, Radeny S, Government of Kenya. A multi sectorial approach to providing reproductive

health information and services to young people in Western Kenya: the Kenya adolescent reproductive health project, FRONTIERS Final Report. Washington: Population Council; 2004 [cited 2013 12 11]. Available from: <https://pdf.usaid.gov/pdf_docs/Pnada289.pdf>

Askew I, Diop NJ. Scaling-up successful youth interventions: examples from Kenya and Senegal. 2007 [cited 2014 Jun 06]. Available from: <https://www.iywg.org/sites/iywg/files/askew-diop_scaling_up.pdf>

Askew I, Evelia H. Mainstreaming and scaling-up the Kenya adolescent reproductive health project.

Washington, DC: Population Council, Frontiers in Reproductive Health. 2007 [cited 2014 Jun 02]. Available from: [www.expandnet.net/P*/KARHP_scaleup_process.pdf](http://www.expandnet.net/PDFs/KARHP_scaleup_process.pdf)

Awofeso N, Rammohan A. Three decades of the integrated child development services program in

India: Progress and problems. In K. Smigorski, editor. Health management – Different approaches and solutions. Rijeka, Croatia: In Tech Open; 2011. [cited 2013 Dec 22]. p. 243-258. Available from: <https://www.intechopen.com/download/pdf/24990>

Binagwaho A, Ngabo F, Wagner CM, Mugeni C, Gatera M, Nutt CT, Nsanzimana S. Integration of comprehensive women's health programmes into health systems: cervical cancer prevention, care and control in Rwanda. Bull World Health Org. 2013; 91(9):697–703. Available from: <http://dx.doi.org/10.2471/BLT.12.116087>

Binagwaho A, Wagner CM, Gatera M, Karema C, Nutt CT, Ngabo F. Achieving high coverage in Rwanda's national human papillomavirus vaccination programme. Bull World Health Org. 2012; 90(8):623–628. doi:10.2471/BLT.11.097253

BRAC. BRAC in Afghanistan: quietly making large impact. 2014 [cited 2014 Aug 14]. Available from:

<http://blog.brac.net/2010/07/brac-in-afghanistan-quietly-making-large-impact/>

Campbell H, Gove S. Integrated management of childhood infections and malnutrition: a global initiative. Arch Dis Child Educ. 1996; 75(6):468-471.

Chanda E, Mukonka VM, Kamuliwo M, Macdonald MB, Haque U. Operational scale entomological intervention for malaria control: Strategies, achievements and challenges in Zambia. Malar J. 2013; 12:10. Available from: <http://www.malariajournal.com/content/12/1/10>

Chandra-Mouli V, Mapella E, John T, Gibbs S, Hanna C, Kampatible N, et al. Standardizing and scaling-up quality adolescent friendly health services in Tanzania. BMC Public Health. 2013; 13(579). Available from: <http://www.biomedcentral.com/1471-2458/13/579>

Chizema-Kawesha E, Miller JM, Steketee RW, Mukonka VM, Mukuka C, Mohamed AD, et al. (2010). Scaling-up malaria control in Zambia: progress and impact 2005-2008. Am J Trop Med Hyg. 2010; 83(3):480-488. doi:10.4269/ajtmh.2010.10-0035

Chowdhury AMR, Alam MA, Ahmed J. Development knowledge and experience – from Bangladesh to Afghanistan and beyond. Bull World Health Org. 2006; 84(4):677-681.

Crosbie E, Sebrie EM, Glantz SA. Strong advocacy led to successful implementation of smokefree

Mexico City. Tob Control. 2010; 20(1):64-72. doi:10.1136/tc.2010.037010

de la Vega ES. Warmi II: building bridges between the community and the health services with a gender and intercultural approach. Washington, DC: Artes Graficas Sagitario; 2008 [cited 2014 Jul 28]. Available from: <http://new.paho.org/hq/dmdocuments/2008/warmi%20ingles.pdf>

Division of Reproductive Health, Ministry of Health. Adolescent and youth sexual and reproductive health evidence-based interventions in Kenya; 2013 [cited 2014 Mar 25]. Available from:

<http://www.fhi360.org/sites/default/files/media/documents/youth-sexual-reproductive-health-interventions-kenya.pdf>

Do TH, Pham TN, Nguyen KT, Vu QN, Nguyen TT, Do TTN, et al. An assessment of the need for contraceptive introduction in Viet Nam. Geneva: WHO; 1995 [cited 2014 May 22]. Available from:

<http://www.who.int/reproductivehealth/publications/family_planning/HRP_ITT_95_3/en/>

Drope J, Glantz S. British Columbia capital regional district 100% smokefree bylaw: a successful public health campaign despite industry opposition. Tob Control. 2003; 12(3):264-268. doi:10.1136/tc.12.3.264

Evelia H, Wanjiur M, Obare F, Birungi H. Ten years of the Kenya adolescent reproductive health

project: what has happened? Nairobi, Kenya: APHIA II OR Project in Kenya / Population Council; 2011 [cited 2014 May 11]. Available from: <http://www.popcouncil.org/uploads/pdfs/2011RH_KARHP10Years.pdf>

Evelia H, Nyambane J, Birungi H, Askew I, Trangsrud R, Muthuuri E, et al. From pilot to program: Scaling-up the Kenya Adolescent Reproductive Health Project. FRONTIERS Final Report. Washington, DC: Population Council; 2008 [cited 2014 Feb 12]. Available from: <http://www.popcouncil.org/uploads/pdfs/frontiers/FR_FinalReports/Kenya_KARHPScalingUp.pdf>

Fajans P, Nguyen TT, Whittaker M, Satia J, Tran TPM, Trinh DC, et al. Strategic choices in scaling-up: introducing injectable contraception and improving quality of care in Viet Nam. In Simmons R, Fajans P, Ghiron L, editors. Scaling-up health service delivery: from pilot innovations to policies and programmes. Geneva: World Health Organization Press; 2007 [cited 2013 Nov 10]. p.31-51. Geneva: WHO. Available from: <http://www.who.int/reproductivehealth/publications/strategic_approach/9789241563512/en/>

FHI 360, PROGRESS & Ministry of Health, Kenya. Adolescent and youth sexual reproductive health: taking stock in Kenya; 2011 [cited 2014 Mar 20]. Available from: <http://www.fhi360.org/sites/default/files/media/documents/youth-sexual-reproductive-health-kenya-phase1.pdf>

Fizzell J, Campbell-Lloyd S, Hallett AJ, Lowbridge CP, Meijer D, Tyner SE. Pandemic (H1N1) 2009 influenza vaccine roll-out in NSW. N S W Public Health Bull. 2010; 21(1-2):32-35.

Fong GT, Hammond D, Hitchman SC. The impact of pictures on the effectiveness of tobacco warnings. Bull World Health Org. 2009; 87(8):640-643. doi:10.2471/BLT.09.069575

Friedland G, Harries A, Coetzee D. Implementation issues in tuberculosis/HIV program collaboration and integration: 3 case studies. J Infect Dis. 2007; 196(Suppl 1), S114-S123. doi:10.1086/518664

Ghosh S. Integrated child development services programme - need for reappraisal. Indian Pediatr. 1997; 34:911-918.

Gonzales F, Arteaga E, Howard-Grabman L. (1998). Scaling-up the WARMI project: lessons learned. In Burkalter BV, Graham VL, editors. Presented papers: High impact PVO child survival programs, Vol. 2. Proceedings of an Expert Consultation, Gallaudet University, 21-24 June 1998. Arlington, VA. 1998 [cited 2014 Mar 19]. Available from: <http://www.eldis.org/vfile/upload/1/document/0708/DOC15246.pdf>

Gloyd S, Montoya P, Floriano F, Chadreque MC, Pfeiffer J, Gimbel-Sherr K. Scaling-up antenatal syphilis screening in Mozambique: transforming policy to action. Sex Transm Dis. 2007; 34(Suppl 7):S31-S36. doi:10.1097/01.olq.0000264586.49616.72

Gove S. Integrated management of childhood illness by outpatient health workers: technical basis and overview. Bull World Health Org. 1997; 75(Supp 1):7-24.

Graff M. Kenya's adolescent reproductive health and development policy: implementation progress and barriers. 2013 [cited 2014 Jun 21]. Available from: <http://www.prb.org/Publications/Articles/2013/kenya-policy-assessment-report.aspx>

Hanson K, Nathan R, Marchant T, Mponda H, Jones C, Bruce J, Stephen G, et al. Vouchers for scaling-up insecticide-treated nets in Tanzania: methods for monitoring and evaluation of a national health system intervention. BMC Public Health. 2008; 8(1). doi:10.1186/1471-2458-8-205

Harries AD, Gomani P, Teck R, de Teck OA, Bakali E, Zachariah R, Libamba E, et al. Monitoring the response to antiretroviral therapy in resource-poor settings: the Malawi model. Trans R Soc Trop Med Hyg. 2004; 98(12):695-701.

Hoek J, Wilson N, Allen M, Edwards R, Thomson G, Li J. (2010). Lessons from New Zealand's introduction of pictorial health warnings on tobacco packaging. Bull World Health Org. 2010; 88(11):861-866. doi:10.2471/BLT.10.076695

Huicho L, Davila M, Campos M, Drasbek C, Bryce J, Victora CG. Scaling-up integrated management of childhood illness to the national level: achievements and challenges in Peru. Health Policy Plan. 2005a; 20(1):14-24. doi:10.1093/heapol/czi002

Huicho L, Davila M, Gonzales F, Drasbek C, Bryce J, Victora C. Implementation of the integrated

management of childhood illness strategy in Peru and its association with health indicators: an

ecological analysis. Health Policy Plan. 2005b; 20(suppl 1):i32-i41.

Joyce S, Askew I, Diagne AF, Diop N, Evelia H. Multisectoral youth RH interventions: the scale-up process in Kenya and Senegal. Washington, DC: The Population Council. 2008 [cited 2014 Apr 09]. Available from: <http://www.popcouncil.org/uploads/pdfs/frontiers/pbriefs/PB13.pdf>

Kapil U. Integrated child development services (ICDS) scheme: a program for holistic development of children in India. Int J Pediatr. 2002; 69(7):597-601.

Kaufman J, Erli Z, Zhenming X. Quality of care in China: Scaling-up a pilot project into a national reform program. Stud Fam Plann. 2006; 37(1):17-28.

Kaufman J, Erli Z, Zhenming X. Strategic choices in scaling-up: Introducing injectable contraception and improving quality of care in Viet Nam. In Simmons R, Fajans P, Ghiron L, editors. Scaling-up health service delivery: from pilot innovations to policies and programmes. Geneva: World Health Organization Press; 2007 [cited 2013 Nov 10]. p. 53-70. Available from: <http://www.who.int/reproductivehealth/publications/strategic_approach/9789241563512/en/>

Kumwenda M, Tom S, Chan AK, Mwinjiwa E, Sodhi S, Joshua M, van Lettow M. Reasons for accepting or refusing HIV services among tuberculosis patients at a TB-HIV integration clinic in Malawi. Int J Tuberc Lung Dis. 2011; 15(12):1663-1668. Available from: <http://dx.doi.org/10.5588/ijtld.10.0741>

Libamba E, Makombe S, Harries AD, Chimzizi R, Salaniponi FM, Schouten EJ et al. Scaling-up antiretroviral therapy in Africa: learning from tuberculosis control programmes - the case of Malawi. Int J Tuberc Lung Dis. 2005; 9(10):1062-1071.

Libamba E, Makombe SD, Harries AD, Schouten EJ, Yu JK, Pasulani O, et al. Malawi's contribution to "3 by 5": achievements and challenges. Bull World Health Org. 2007; 85(2):156-160.

Libamba E, Makombe S, Mhango E, de Ascurra Teck O, Limbambala E, Schouten E, et al. Supervision, monitoring and evaluation of nationwide scale-up of antiretroviral therapy in Malawi. Bull World Health Org. 2006; 84(4):320-326.

Magesa SM, Lengeler C, deSavigny D, Miller JE, Njau RJA, Kramer K, et al. Creating an "enabling environment" for taking insecticide treated nets to national scale: the Tanzanian experience. Malar J. 2005; 4:34. doi:10.1186/1475-2875-4-34

Makombe SD, Jahn A, Tweya H, Chuka S, Yu JK, Hochgesang M, et al. A national survey of the impact of rapid scale-up of antiretroviral therapy on health-care workers in Malawi: effects on human resources and survival. Bull World Health Org. 2007; 85(11):851-857. doi: 10.2471/BLT.07.041434

Ministry of Health, Malawi. Treatment of AIDS: Guidelines for the use of antiretroviral therapy in Malawi (3^rd^ ed.). 2003 [cited 2014 Mar 02]. Available from: <http://www.who.int/hiv/ARV-guidelines.pdf>

Ministry of Health, Malawi. Treatment of AIDS: Guidelines for the use of antiretroviral therapy in Malawi (3^rd^ ed.). 2008 [cited 2014 Mar 02]. Available from: <http://apps.who.int/medicinedocs/documents/s18803en/s18803en.pdf>

National Council for Population & Development (NCPD), Division of Reproductive Health (DRH), & Population Reference Bureau (PRB). Kenya adolescent reproductive health and development policy: implementation assessment report. USAID. 2013 [cited 2014 Apr 27]. Available from: [www.prb.org/pdf13/kenya-policy-assessment-report.pdf](http://www.prb.org/pdf13/kenya-policy-assessment-report.pdf)

National Tuberculosis Control Programme, MOH; HIV/AIDS Unit, Department of Clinical Services, MOH; National AIDS Commission. Report of a country-wide survey of HIV/AIDS services in Malawi. 2004 [cited 2014 Aug 02]. Available from: <https://www.globalhivmeinfo.org/Gamet/pdf/682_Survey%20of%20HIVAIDS%20services%20in%20Malawi%202003.pdf>

National Tuberculosis Control Programme, MOH; HIV/AIDS Unit, Department of Clinical Services, MOH; National AIDS Commission, Lilongwe; & Centers for Disease Control and Prevention, Malawi. (2005). Report of a country-wide survey of HIV/AIDS services in Malawi. 2005 [cited 2014 Apr 09]. Available from: <http://www.who.int/hiv/Situational-analysis-05.pdf>

Njau RJA, de Savigny D, Gilson L, Mwageni E, Mosha FW. Implementation of an insecticide-treated net subsidy scheme under a public-private partnership for malaria control in Tanzania - challenges in implementation. Malar J. 2009; 8: 201. doi:10.1186/1475-2875-8-201

Nguyen TT, Do TH, Vu QN, Do TN, Whittaker M, Fajans P, et al. The strategic approach to the introduction of DMPA as an opportunity to improve quality of care for all contraceptive methods in Viet Nam. Asia Pac Popul J. 2000; 15(4): 63-86.

Obasi AI, Cleophas B, Ross DA, Chima KL, Mmassy G, Gavyole A, et al. Rationale and design of the MEMA kwa Vijana adolescent sexual and reproductive health intervention in Mwanza Region, Tanzania. AIDS Care. 2006; 18(4):311-322. doi:10.1080/09540120500161983

Pan American Health Organization (PAHO). 2014. Implementing comprehensive childhood health care in Peru. Geneva: WHO; 2014 [cited 2014 Aug 11]. Available from: <http://www.paho.org/pahousaid/index.php?option=com_content&view=article&id=5344:peru-implementing-comprehensive-childhood-health-care-peru&Itemid=3455&lang=en>

Pan American Health Organization (PAHO). WARMI II: building bridges between the community and the health service with a gender and cultural approach. Geneva: WHO; 2008 [cited 2014 Mar 26]. Available from: <http://webcache.googleusercontent.com/search?q=cache:0A0s9-nxpswJ:https://healthresearchweb.org/%3Faction%3Ddownload%26file%3Dethnicity_health_best_practices.pdf+&cd=1&hl=en&ct=clnk&gl=ca&client=safari>

Passmore JW, Nguyen LH, Nguyen NP, Olive J. The formulation and implementation of a national helmet law: a case study from Viet Nam. Bull World Health Org. 2010; 88(10):783-787. doi:10.2471/BLT.09.071662

Pervin A, Passmore J, Sidik M, McKinley T, Nguyen THT, Nguyen PN. Viet Nam's mandatory motorcycle helmet law and its impact on children. Bull World Health Org. 2009; 87(5):369-373. doi:10.2471/BLT.08.057109

Population Council. Life-skills education leads to improved effect on adolescents' sexual behavior. Nairobi, Kenya: Population Council; 2010 [cited 2014 May 13]. Available from: [www.popcouncil.org/uploads/pdfs/2010RH_APHIAII_ORSum3.pdf](http://www.popcouncil.org/uploads/pdfs/2010RH_APHIAII_ORSum3.pdf)

PSI Malaria Control. SMARTNET: a Tanzanian public-private partnership to prevent malaria. 2005 [cited 2014 Apr 19]. Available from: [http://www.shopsproject.org/sites/default/files/resources/2815_file_Tz_F_screen.pdf‬‬‬‬‬‬‬‬‬‬‬‬‬‬‬‬‬‬‬‬‬‬‬](http://www.shopsproject.org/sites/default/files/resources/2815_file_Tz_F_screen.pdf)

Rah JH, Garg A, Naidu BRG, Agrawal DD, Pandey RS, Aguayo VM. Reaching the poor with adequately iodized salt through the supplementary nutrition programme and midday meal scheme in Madhya Pradesh, India. Bull World Health Org. 2013; 91(7):S40-S44.

Rao N. Children's rights to survival, development, and early education in India: the critical role of the integrated child development services program. Int J Early Child. 2005; 37(3):15-31.

Reichenbach L, Shimul SN. Sustaining health: The role of BRAC's community health volunteers in Bangladesh, Afghanistan and Uganda. Research Monograph Series No. 49. 2011 [cited 2014 Jul 01]. Available from: <http://research.brac.net/monographs/Monograph_49.pdf>

Renju J, Andrew B, Nyalai K, Kishamawe C, Kato C, Changalucha J, et al. A process evaluation of the scale-up of a youth-friendly health services initiative in northern Tanzania. J Int AIDS Soc. 2010a; 13(32). Available from: <http://www.jiasociety.org/content/13/1/32>

Renju J, Makokha M, Kato C, Medard L, Andrew B, Remes P, et al. Partnering to proceed: scaling-up adolescent sexual reproductive health programmes in Tanzania. Health Res Policy Syst. 2010b; 8(12):28-40. doi:10.1186/1478-4505-8-12

Renju J, Nyalali K, Andrew B, Kishamawe C, Kimaryo M, Remes P, et al. Scaling-up a school-based sexual and reproductive health intervention in rural Tanzania: a process evaluation describing the implementation realities for the teachers. Health Educ Res. 2010c; 25(6):903-916. doi:10.1093/her/cyq041

Roemer R, Taylor A, Lariviere J. Origins of the WHO framework convention on tobacco control. Am J Public Health. 2005; 95(6):936-938. doi:10.2105/AJPH.2003.025908

Seshadri SR. (2003). Constraints to scaling-up health programmes: a comparative study of two Indian states. J Int Dev. 2003; 15(1):101-114. doi:10.1002/jid.968

Shibuya K, Ciecierski C, Guindon E, Bettcher DW, Evans DB, Murray CJL. WHO Framework convention on tobacco control: development of an evidence based global public health treaty. BMJ. 2003; 327(7407):154-7.

Simmons R, Hall P, Diaz J, Diaz M, Fajans P, Satia J. The strategic approach to contraceptive introduction. Stud Fam Plann. 1997; 28(2):79-94.

Spicehandler J, Simmons R. Contraceptive introduction reconsidered: a review and conceptual framework. Geneva: WHO; 1994 [2014 Mar 08]. Available from: <http://www.who.int/reproductivehealth/publications/family_planning/HRP_ITT_94_1/en/>

Steketee RW, Sipilanyambe N, Chimumbwa, J, Banda JJ, Mohamed A, Miller J. et al. National malaria control and scaling-up for impact: the Zambia experience through 2006. Am J Trop Med Hyg. 2008; 79(1):45-52.

Tulloch J. Integrated approach to child health in developing countries. Lancet. 1999; 354(Suppl II):16-20.

Ved R. Scaling-up ICDS: can universalisation address persistent malnutrition? IDS Bull. 2009; 40(4):53-59.

Wamburi AO. Sustaining adolescents & reproductive health in Kenya through a multi-sectorial program approach. USAID, PATH; unknown [cited 2014 Jan 17]. Available from: <http://dev13.jhsph.nts.jhu.edu/research/centers-and-institutes/bill-and-melinda-gates-institute-for-population-and-reproductive-health/_pdf/policy_practice/adolhealth/presentations/C_Session/PPT/2C_Wamburi_sustaining_adolscents_rep_health.pdf>

Wang C. History of the Chinese family planning program: 1970-2010. Contracept. 2012; 85:563-569. doi: 10.1016/j.contraception.2011.10.013

World Health Organization (2003). WHO framework convention on tobacco control. Geneva: WHO; 2003 [cited 2013 Nov 24]. Available from: <http://whqlibdoc.who.int/publications/2003/9241591013.pdf>

World Health Organization. The WHO strategic approach to strengthening sexual and reproductive health policies and programmes. Geneva: WHO; 2007 [cited 2014 Sept 08]. Available from: <http://whqlibdoc.who.int/hq/2007/WHO_RHR_07.7_eng.pdf?ua=1>

Yothasamut J, Putchong C, Sirisamutr T, Teerawattananon Y, Tantivess S. Scaling up cervical cancer screening in the midst of human papillomavirus vaccination advocacy in Thailand. BMC Health Ser Res. 2010; 10(Suppl 1), S5. Available from: https://doi.org/10.1186/1472-6963-10-S1-S5
