# Supplementary material for: Developing a framework to inform scale-up success for population health interventions: a critical interpretive synthesis of the literature
Source: Glob Health Res Policy. 2020 Apr 29;5:18. doi: 10.1186/s41256-020-00141-8 (PMC7189598; doi:10.1186/s41256-020-00141-8)
Supplement: Supplementary file 7 — Additional file 7: Summary of the retained literature ( n = 77). [file 41256_2020_141_MOESM7_ESM.docx]

Table 1: Summary of the retained literature (*n*=77)

*Legend.*

**Symbols defined:** # = Number; * = County income level obtained from: <http://data.worldbank.org/country>; ✪ = scale-up success status, explicitly stated by the author; ★ = scale-up success status, based on author descriptions

**Acronyms and short-hand defined (in alphabetical order):** ART = Antiretroviral; DMPA = Depo-Provera®; GL = grey literature; HIV = human immunodeficiency virus; Implement. = implementation; MOH = Ministry of Health; NGOs = non-government organizations; PHI= population health intervention; pop. = population; PRL = peer-reviewed literature; supp. docs. = supplementary documents; Sustain. = sustained; TB = tuberculosis

| Sample Information | | |  | PHI Characteristics | | |  | Scale-up Characteristics | |
| --- | --- | --- | --- | --- | --- | --- | --- | --- | --- |
| PHI # | Primary literature (supp. docs.) | # of PRL (GL) |  | Country (pop. focus) Income* | PHI Health Focus | PHI description |  | Scale-up approach | Scale-up Implement. (Sustain.) |
| 1 | Binagwaho et al., 2012 (Binagwaho et al., 2013) | 2 (0) |  | Rwanda (girls of appropriate age, starting in grade 6 of primary school) Low-income country | Cervical cancer prevention | This human papilloma virus (HPV) vaccination strategy is part of a larger, multi-component PHI called Rwanda's National Strategic Plan for the Prevention, Control, and Management of Cervical Lesions and Cancer |  | Rwanda's Ministry of Health formed a public-private community partnership with Merck to offer Gardasil HPV vaccine and included various ministries; Received technical assistance from international organizations and universities; Media sensitization campaigns were set-up in advance to inform the public and communication was made with local leaders | Successful✪ (Unknown) |

Table 1 continued

| Sample Information | | |  | PHI Characteristics | | |  | Scale-up Characteristics | |
| --- | --- | --- | --- | --- | --- | --- | --- | --- | --- |
| PHI # | Primary literature (supp. docs.) | # of PRL (GL) |  | Country (pop. focus) Income* | PHI Health Focus | PHI description |  | Scale-up approach | Scale-up Implement. (Sustain.) |
| 2 | Chowdhury et al., 2006 (BRAC, 2014; Reichenbach & Shimul, 2011) | 1 (2) |  | Afghanistan (21 of 34 provinces) Low-income country | Health, education, microfinance, women's empowerment, agriculture, capacity development, and local government strengthening | Holistic approach to alleviate poverty and tackle its root causes; Programmes in microfinance, health (i.e., maternal and newborn health, child health and immunization, nutrition, communicable diseases, mental health, disability, and supply of essential drugs), education, capacity building and training, agriculture, and the National Solidarity Program |  | Training community workers and teachers, Close NGO-government collaboration; Social mobilization and community participation; Identified potential challenges to scale-up; Setting up health posts and schools; Recruitment of microfinance borrowers; Established two training and resource centres, with residential facilities for 150 trainees; Established a research unit | Successful ✪ (Successful ✪) |

Table 1 continued

| Sample Information | | |  | PHI Characteristics | | |  | Scale-up Characteristics | |
| --- | --- | --- | --- | --- | --- | --- | --- | --- | --- |
| PHI # | Primary literature (supp. docs.) | # of PRL (GL) |  | Country (pop. focus) Income* | PHI Health Focus | PHI description |  | Scale-up approach | Scale-up Implement. (Sustain.) |
| 3 | Crosbie et al., 2010 | 1 (0) |  | Mexico (population of Mexico City) Middle-income country | Public smoking ban | Ratifying the WHO Framework Convention for Tobacco Control and Implementing municipal smokefree legislation |  | Forming advocacy groups; Staging media campaigns; Strong political leadership to support smoking bans; Enforcing compliance through inspections and fines | Successful ✪ (Partially successful✪) |
| 4 | Drope & Glantz, 2003 (Roemer et al., 2005; Shibuya et al., 2003; WHO, 2003) | 3 (1) |  | Canada (population of Victoria) High-income country | Public smoking ban | A 100% smokefree bylaw in all public places in the British Columbia Capital Regional District |  | Comprehensive communications strategy including media and education campaigns to counter tobacco industry rhetoric and understand the new bylaw, well-written bylaws, and persistent enforcement | Successful ✪ (Successful ✪) |

Table 1 continued

| Sample Information | | |  | PHI Characteristics | | |  | Scale-up Characteristics | |
| --- | --- | --- | --- | --- | --- | --- | --- | --- | --- |
| PHI # | Primary literature (supp. docs.) | # of PRL (GL) |  | Country (pop. focus) Income* | PHI Health Focus | PHI description |  | Scale-up approach | Scale-up Implement. (Sustain.) |
| 5 | Evelia et al., 2008 (Askew & Diop, 2007; Askew & Evelia, 2007; Askew et al., 2004; Evelia et al, 2011; Graff, 2013; Joyce et al., 2008; FHI 360, 2011; Div. of Reproductive Health, 2013; Population Council, 2010; Wamburi, unknown; NCPD et al., 2013) | 0 (12) |  | Kenya (youths 10-20 years-old) Middle-income country | Reproductive health project | Provided reproductive health education program and adolescent friendly services |  | Collaborations, multi-stakeholder involvement; Improving support and promoting dialogue within communities, com-munity outreach; Monitoring and evaluation; Integrated into three existing government structures, net-works, and systems at the district level; Operations research, surveys; Adaptation; Institutionalization, Expansion; Replication; Technical assistance; Training peer educators; Advocacy, mobilization activities, lobbying; Budgeting; Initiate inter-ministerial committees at various levels; Buy-in; Communication | Successful ✪ (Successful ✪) |

Table 1 continued

| Sample Information | | |  | PHI Characteristics | | |  | Scale-up Characteristics | |
| --- | --- | --- | --- | --- | --- | --- | --- | --- | --- |
| PHI # | Primary literature (supp. docs.) | # of PRL (GL) |  | Country (pop. focus) Income* | PHI Health Focus | PHI description |  | Scale-up approach | Scale-up Implement. (Sustain.) |
| 6 | Fajans et al., 2007 (Do et al., 1995; Nguyen et al., 2000; Simmons et al., 1997; Spicehandler & Simmons, 1994; WHO, 2007) | 2 (4) |  | Viet Nam (females of reproductive age) Middle-income country | Reproductive health / Family planning | Provide injectable contraceptive DMPA as part of a package of interventions to improve quality of care in the provision of all contraceptives in Vietnamese family planning |  | Phased introduction; Developed tool kit w/guidelines and materials; Orientation workshops; Training of providers; Supervision and monitoring; Development of national standards; Policy advocacy and info dissemination | Partially successful✪ (Partially successful✪) |
| 7 | Fizzell et al., 2010 | 1 (0) |  | Australia (population of New South Wales) High-income country | H1N1 vaccination | Access to HIN1 vaccination |  | Rollout using a general practitioner and Aboriginal Health Service-based model for delivery of the vaccine | Successful ★ (Not applicable) |

Table 1 continued

| Sample Information | | |  | PHI Characteristics | | |  | Scale-up Characteristics | |
| --- | --- | --- | --- | --- | --- | --- | --- | --- | --- |
| PHI # | Primary literature (supp. docs.) | # of PRL (GL) |  | Country (pop. focus) Income* | PHI Health Focus | PHI description |  | Scale-up approach | Scale-up Implement. (Sustain.) |
| 8 | Friedland et al., 2007 (Kumwenda et al., 2011) | 2 (0) |  | Malawi (national) Low-income country | Integrated care for TB and HIV | Improve collaboration between HIV/AIDS and TB programs; Expand HIV voluntary counseling and testing (VCT) services for patients with TB and the general public; Provide cotrimoxazole preventive therapy to HIV-infected patients with TB; and Provide Antiretroviral therapy (ART) to patients with AIDS, including those with HIV-associated TB |  | Phased countrywide implementation of HIV testing and cotrimoxazole therapy for all patients with TB in all 60 facilities in public sector; Equitable access to free antiretroviral drugs; Drug procurement; Increased collaboration between TB and HIV programs; Staff training; Initiating ART in new patients; Registering and monitoring case numbers and treatment outcome | Successful ✪ (Partially successful★) |

Table 1 continued

| Sample Information | | |  | PHI Characteristics | | |  | Scale-up Characteristics | |
| --- | --- | --- | --- | --- | --- | --- | --- | --- | --- |
| PHI # | Primary literature (supp. docs.) | # of PRL (GL) |  | Country (pop. focus) Income* | PHI Health Focus | PHI description |  | Scale-up approach | Scale-up Implement. (Sustain.) |
| 9 | Gloyd et al., 2007 | 1 (0) |  | Mozambique (women in antenatal care) Low-income country | Maternal and prenatal health | Integrating syphilis screening in antenatal care; Strengthened health system; Changed from rapid plasma reagin (RPR) to immunochromato-graphic strip (ICS) so that it can provide access to testing for patients in peripheral facilities without laboratories and improves testing |  | Persistent advocacy at all levels of the Ministry of Health; Community involvement and mobilization; Screening integrated into the National Health System; Control and provision of supply shortages and stock outs; Continuous monitoring, evaluation, and supervision; Intensive supervision until appropriate habits are formed; Ensuring adequate facilities | Successful ✪ (Successful ✪) |

Table 1 continued

| Sample Information | | |  | PHI Characteristics | | |  | Scale-up Characteristics | |
| --- | --- | --- | --- | --- | --- | --- | --- | --- | --- |
| PHI # | Primary literature (supp. docs.) | # of PRL (GL) |  | Country (pop. focus) Income* | PHI Health Focus | PHI description |  | Scale-up approach | Scale-up Implement. (Sustain.) |
| 10 | Gonzales et al., 1998 (de la Vega, 2008; PAHO, 2008) | 0 (3) |  | Bolivia (women and infants in 513 rural communities) Middle-income country | Maternal and perinatal health | Gender sensitive participatory methodology, teaching women how to participate and mobilize their community and learn about reproductive health and how to obtain access to quality health care Build links between the community and health service facilities with a gender, intercultural and community participation approach |  | Develop, implement and document a successful demonstration project; Disseminate project methods and results; Advocate to build consensus and influence policy; Mobilize resources; Define organizational structure and philosophy of the national project; Establish agreements with partners (MOH and NGOs); Provide training and technical assistance; Coordinating activities with partner agencies; Develop and use monitoring and evaluation systems; Integrated in municipal governments' annual plans | Successful ✪ (Successful ✪) |

Table 1 continued

| Sample Information | | |  | PHI Characteristics | | |  | Scale-up Characteristics | |
| --- | --- | --- | --- | --- | --- | --- | --- | --- | --- |
| PHI # | Primary literature (supp. docs.) | # of PRL (GL) |  | Country (pop. focus) Income* | PHI Health Focus | PHI description |  | Scale-up approach | Scale-up Implement. (Sustain.) |
| 11 | Hoek et al., 2010 (Fong et al., 2009) | 2 (0) |  | New Zealand (national) High-income country | Tobacco control | Improve knowledge of smoking harms and cessation-related behaviours; Selection of images |  | Running integrated mass media campaigns for Quitline and the introduction of pictorial warning labels on tobacco packages; Prepared online fact sheets regarding smoking and disease outcomes; Policy implementation; Anticipating and preparing for adverse responses to the PHI; Monitoring; Obtained guidance from Australian counterparts who already had pictorial warnings on tobacco packages | Successful ✪ (Successful ★) |

Table 1 continued

| Sample Information | | |  | PHI Characteristics | | |  | Scale-up Characteristics | |
| --- | --- | --- | --- | --- | --- | --- | --- | --- | --- |
| PHI # | Primary literature (supp. docs.) | # of PRL (GL) |  | Country (pop. focus) Income* | PHI Health Focus | PHI description |  | Scale-up approach | Scale-up Implement. (Sustain.) |
| 12 | Huicho et al., 2005a (Campbell & Gove, 1996; Huicho et al., 2005b; Gove, 1997; PAHO, 2014; Tulloch, 1999) | 5 (1) |  | Peru (children under 5 years-old) Middle-income country | Child health | Improving health worker performance; Improving health system support; and Improving family and community practice |  | Scaled up parallel to existing child health programmes; Was later institutionalized by the Ministry of Health as one of the strategies of the Comprehensive Childhood Health Care Model | Partially successful✪ (Partially successful★) |
| 13 | Kaufman et al., 2007 (Kaufman et al., 2006; Wang, 2012) | 2 (1) |  | China (national) Middle-income country | Reproductive health / Family planning | Reorienting the national family planning programme; Greater focus on patient needs, providing informed choice of contraceptives, and better quality services |  | Providing local ownership; Careful selection of pilot sites; Mobilizing political networks; Cultivating and educating allies in senior leadership positions; Advocacy; Customized, context-specific requirements; Strategic use of donor funding and technical assistance | Successful ✪ (Successful ★) |

Table 1 continued

| Sample Information | | |  | PHI Characteristics | | |  | Scale-up Characteristics | |
| --- | --- | --- | --- | --- | --- | --- | --- | --- | --- |
| PHI # | Primary literature (supp. docs.) | # of PRL (GL) |  | Country (pop. focus) Income* | PHI Health Focus | PHI description |  | Scale-up approach | Scale-up Implement. (Sustain.) |
| 14 | Libamba et al., 2006 (Harries et al., 2004; Libamba et al., 2005; Libamba et al., 2007; Makombe et al., 2007; Ministry of Health Malawi, 2003; 2008; National Tuberculosis Control Programme, 2004; 2005) | 5 (4) |  | Malawi (national) Low-income country | Antiretroviral (ART) drug therapy | Increased access to ART drug therapy; Prepared national ART guidelines; Standardized treatment outcomes |  | Capacity building, including classroom training, formal examination, and clinical attachments at experienced sites already delivering ART; Formal readiness assessment at each clinic, competence must be demonstrated for each line of regimen; Supervisory visits; Prepared detailed plans for ART services; Routine monitoring and evaluation; Effective procurement of drugs; Regular communication amongst stakeholders | Successful ✪ (Successful ★) |

Table 1 continued

| Sample Information | | |  | PHI Characteristics | | |  | Scale-up Characteristics | |
| --- | --- | --- | --- | --- | --- | --- | --- | --- | --- |
| PHI # | Primary literature (supp. docs.) | # of PRL (GL) |  | Country (pop. focus) Income* | PHI Health Focus | PHI description |  | Scale-up approach | Scale-up Implement. (Sustain.) |
| 15 | Magesa et al., 2005 (Hanson et al., 2008; Njau et al., 2009; PSI Malaria Control, 2005) | 3 (1) |  | Tanzania (national) Low-income country | Malaria control and prevention | Providing accessible and affordable insecticide-treated nets (ITN) |  | Multi-stakeholder action; Coordinated public-private alliance; Creating an enabling environment; Promoting the development of a commercial sector for ITN; Developing a national voucher scheme; Research; Procurement of supplies; Providing consumer protection and addressing regulatory issues; Advocacy, lobbying, negotiating, and social marketing; Successful funding applications; Budgeting; Monitoring and evaluation | Successful ✪ (Successful ★) |

Table 1 continued

| Sample Information | | |  | PHI Characteristics | | |  | Scale-up Characteristics | |
| --- | --- | --- | --- | --- | --- | --- | --- | --- | --- |
| PHI # | Primary literature (supp. docs.) | # of PRL (GL) |  | Country (pop. focus) Income* | PHI Health Focus | PHI description |  | Scale-up approach | Scale-up Implement. (Sustain.) |
| 16 | Passmore et al., 2010 (Pervin et al., 2009) | 2 (0) |  | Viet Nam (national) Middle-income country | Road safety | Strengthened existing motorcycle helmet law and helmet wearing requirements |  | Increased penalties and stringent police enforcement; Intensive advanced public education; Continual monitoring of legislation; Identified and addressed loopholes detrimental to effectiveness; Inter-sectorial collaboration and advocacy; Improved availability and quality of helmets | Successful ✪ (Successful ★) |
| 17 | Renju et al., 2010a (Chandra-Mouli et al., 2013; Obasi et al, 2006; Renju et al., 2010b; Renju et al., 2010c) | 5 (0) |  | Tanzania (youth in 4 districts of Mwanza region) Low-income country | Youth friendly health services and education | Provide reproductive health education, youth-friendly reproductive health services, and community-based condom provision |  | Scaled up through existing government health and school structures; Community mobilization; Participatory approach | Successful ✪ (Successful ★) |

Table 1 continued

| Sample Information | | |  | PHI Characteristics | | |  | Scale-up Characteristics | |
| --- | --- | --- | --- | --- | --- | --- | --- | --- | --- |
| PHI # | Primary literature (supp. docs.) | # of PRL (GL) |  | Country (pop. focus) Income* | PHI Health Focus | PHI description |  | Scale-up approach | Scale-up Implement. (Sustain.) |
| 18 | Steketee et al., 2008 (Chizema-Kawesha et al., 2010; Chanda et al., 2013) | 3 (0) |  | Zambia (national) Middle-income country | Malaria control | Package of malaria prevention and control strategies, including insecticide-treated mosquito nets, indoor residual spraying, intermittent preventive treatment during pregnancy, and prompt effective case management |  | Rapid national scale-up; Commitment by all stakeholders towards one national plan, one coordination mechanism, and one monitoring and evaluation system; Embrace cycle of planning, resourcing, implementing, monitoring, and evaluating; Business planning and financing; Annual planning; Donor support; Advocacy and communication; Campaigns; Consensus building activities; Consultations | Successful ✪ (Successful ✪) |

Table 1 continued

| Sample Information | | |  | PHI Characteristics | | |  | Scale-up Characteristics | |
| --- | --- | --- | --- | --- | --- | --- | --- | --- | --- |
| PHI # | Primary literature (supp. docs.) | # of PRL (GL) |  | Country (pop. focus) Income* | PHI Health Focus | PHI description |  | Scale-up approach | Scale-up Implement. (Sustain.) |
| 19 | Ved, 2009 (Awofeso & Rammohan, 2011; Ghosh, 1997, Kapil, 2002; Rah et al., 2013; Rao, 2005; Seshadri, 2003) | 6 (1) |  | India (children 0-6 years and pregnant and lactating mothers) Middle-income country | Child and maternal health | Centralized, national programs nutrition, development, education, and health services |  | Integrated into government services; Standard implementation approach for all locations; Top-down, rapid expansion without sufficient nutrition and health training for the workers; Weak and unsystematic monitoring and evaluation system | Partially successful✪ (Partially successful✪) |
| 20 | Yothasamut et al., 2010 | 1 (0) |  | Thailand (1 million women 35-60 years old) Middle-income country | Women's reproductive health | Pap smear and visual inspections with acetic acid (VIA) testing services |  | Extending cervical cancer screening services; Sporadic media coverage and information events; Limited stakeholder involvement; Top-down approach | Unsuccessful ✪ (Not applicable) |
